# Supplementary material for: Tailoring lipid management interventions to reduce inequalities in cardiovascular disease risk management in primary care for deprived communities in Northern England: a mixed-methods intervention development protocol
Source: BMJ Open. 2022 Jul 4;12(7):e058951. doi: 10.1136/bmjopen-2021-058951 (PMC9255393; doi:10.1136/bmjopen-2021-058951)
Supplement: Supplementary data [file bmjopen-2021-058951supp001.pdf]

Supplementary Material 1 Search strategy for MEDLINE (Ovid) (1996 to October Week 4 2021)

1. exp Cardiovascular Diseases/
2. cardio*.tw.
3. cardia*.tw.
4. heart*.tw.
5. coronary*.tw.
6. angina*.tw.
7. ventric*.tw.
8. myocard*.tw.
9. pericard*.tw.
10. isch?em*.tw.
11. emboli*.tw.
12. arrhythmi*.tw.
13. thrombo*.tw.
14. atrial fibrillat*.tw.
15. tachycardi*.tw.
16. endocardi*.tw.
17. (sick adj sinus).tw.
18. exp Stroke/

Fu Y, *et al. BMJ Open* 2022; 12:e058951. doi: 10.1136/bmjopen-2021-058951

19. (stroke or strokes).tw.
20. cerebrovasc*.tw.
21. cerebral vascular.tw.
22. apoplexy.tw.
23. ((brain* or cerebral or lacunar) adj2 infarct*).tw.
24. exp Hypertension/
25. hypertensi*.tw.
26. peripheral arter* disease*.tw.
27. ((high or increased or elevated) adj2 blood pressure).tw.
28. exp Hyperlipidemias/
29. hyperlipid*.tw.
30. hyperlip?emia*.tw.
31. hypercholesterol*.tw.
32. hypercholester?emia*.tw.
33. hyperlipoprotein?emia*.tw.
34. hypertriglycerid?emia*.tw.
35. exp Arteriosclerosis/
36. exp Cholesterol/
37. cholesterol.tw.
38. Blood Pressure/

Fu Y, *et al*. *BMJ Open* 2022; 12:e058951. doi: 10.1136/bmjopen-2021-058951

39. 1 or 2 or 3 or 4 or 5 or 6 or 7 or 8 or 9 or 10 or 11 or 12 or 13 or 14 or 15 or 16 or 17 or 18 or 19 or 20 or 21 or 22 or 23 or 24 or 25 or 26 or 27 or 28 or 29 or 30 or 31 or 32 or 33 or 34 or 35 or 36 or 37 or 38
40. exp Socioeconomic Factors/
41. exp social class/
42. socioecon*.tw.
43. demographic*.tw.
44. disadvantage*.tw.
45. disparit*.tw.
46. deprivation.tw.
47. exp Health Services Accessibility/
48. deprive*.tw.
49. pover*.tw.
50. inequalit*.tw.
51. education*.tw.
52. unemploy*.tw.
53. employed.tw.
54. employment.tw.
55. income.tw.
56. occupation*.tw.
57. SES.tw.

58. class.tw.
59. economic.tw.
60. (social adj1 (class or factor or factors)).tw.
61. 40 or 41 or 42 or 43 or 44 or 45 or 46 or 47 or 48 or 49 or 50 or 51 or 52 or 53 or 54 or 55 or 56 or 57 or 58 or 59 or 60
62. exp General Practitioners/
63. exp General Practice/
64. general pract*.tw.
65. exp Family Practice/
66. family pract*.tw.
67. family medicine/
68. exp Family Practice/
69. exp Physicians, Family/
70. family phys*.tw.
71. exp Nurse Practitioners/
72. exp Nurse Clinicians/
73. nurse pract*.tw.
74. nurse clinic*.tw.
75. exp Primary Health Care/
76. primary care.tw.
77. exp Community Health Services/

78. community care.tw.

79. 62 or 63 or 64 or 65 or 66 or 67 or 68 or 69 or 70 or 71 or 72 or 73 or 74 or 75 or 76 or 77 or 78

80. 39 and 61 and 79

81. (Algeria$ or Egypt$ or Liby$ or Morocc$ or Tunisia$ or Western Sahara$ or Angola$ or Benin or Botswana$ or Burkina Faso or Burundi or Cameroon or Cape Verde or Central African Republic or Chad or Comoros or Congo or Djibouti or Eritrea or Ethiopia$ or Gabon or Gambia$ or Ghana or Guinea or Keny$ or Lesotho or Liberia or Madagasca$ or Malawi or Mali or Mauritania or Mauritius or Mayotte or Mozambiq$ or Namibia$ or Niger or Nigeria$ or Reunion or Rwand$ or Saint Helena or Senegal or Seychelles or Sierra Leone or Somalia or South Africa$ or Sudan or Swaziland or Tanzania or Togo or Ugand$ or Zambia$ or Zimbabw$ or China or Chinese or Hong Kong or Macao or Mongolia$ or Taiwan$ or Belarus or Moldov$ or Russia$ or Ukraine or Afghanistan or Armenia$ or Azerbaijan or Bahrain or Cyprus or Cypriot or Georgia$ or Iran$ or Iraq$ or Israel$ or Jordan$ or Kazakhstan or Kuwait or Kyrgyzstan or Leban$ or Oman or Pakistan$ or Palestin$ or Qatar or Saudi Arabia or Syria$ or Tajikistan or Turkmenistan or United Arab Emirates or Uzbekistan or Yemen or Bangladesh$ or Bhutan or British Indian Ocean Territory or Brunei Darussalam or Cambodia$ or India$ or Indonesia$ or Lao or People's Democratic Republic or Malaysia$ or Maldives or Myanmar or Nepal or Philippin$ or Singapore or Sri Lanka or Thai$ or Timor Leste or Vietnam or Albania$ or Andorra or Bosnia$ or Herzegovina$ or Bulgaria$ or Croatia$ or Estonia or Faroe Islands or Greenland or Liechtenstein or Lithuani$ or Macedonia or Malta or maltese or Romania or Serbia$ or Montenegro or Slovenia or Svalbard or Argentina$ or Belize or Bolivia$ or Brazil$ or chile or Chilean or Colombia$ or Costa Rica$ or Cuba or Ecuador or El Salvador or French Guiana or Guatemala$ or Guyana or Haiti or Honduras or Jamaica$ or Nicaragua$ or Panama or Paraguay or Peru or Puerto Rico or Suriname or Uruguay or Venezuela or developing countr$ or south America$).ti,sh. [mp=title, abstract, original title, name of substance word, subject heading word, floating sub-heading word, keyword heading word, organism supplementary concept word, protocol supplementary concept word, rare disease supplementary concept word, unique identifier, synonyms]

82. 80 not 81

83. limit 82 to (abstracts and english language and humans and yr="2011 -Current")

Fu Y, *et al. BMJ Open* 2022; 12:e058951. doi: 10.1136/bmjopen-2021-058951
