# Supplementary material for: Tailoring lipid management interventions to reduce inequalities in cardiovascular disease risk management in primary care for deprived communities in Northern England: a mixed-methods intervention development protocol
Source: BMJ Open. 2022 Jul 4;12(7):e058951. doi: 10.1136/bmjopen-2021-058951 (PMC9255393; doi:10.1136/bmjopen-2021-058951)
Supplement: Supplementary data [file bmjopen-2021-058951supp002.pdf]

Interview topic guide

This interview is to learn from your experience on what and how lipid management is currently delivered to patients in your practice. The aim is to understand the implementation process and resources available for the existing interventions targeting patients with cardiovascular disease (CVD) and/or risks, as well as your needs and any challenges to providing lipid management optimisation in deprived communities.

The interview will be recorded with your permission. It will last for about 45-60 minutes and any information you give will be kept confidential and anonymous.

Our conversation will cover:

- Your job title, an overview of patients in your practice, what your role involves for patients with CVD risks;
- Your understanding of lipid management and what it involves;
- How patients with CVD (or CVD risks) are managed and their clinical pathways in your practice (screening/assessment/monitoring/treatment/outcomes/referrals):
    - How you identify patients with CVD risks (e.g. NHS Health Check; frequency), and is there anything else that would indicate the need for care for you;
    - What assessment tool(s) you use to understand their needs;
    - How you monitor their disease progress;
    - Existing treatment and care provided for patients and rationale for that;
    - Clinically meaningful outcomes;
    - How and when you decide on referrals and for what services.
- Your views on the implementation process of current interventions targeting patients with CVD in your practice, and what resource is needed for their delivery;
- Your experience/challenges of managing patients' needs, and factors that influence the delivery and quality of lipid management to patients;
- The impact of COVID on lipid management in your practice and solutions implemented;
- To what extent you have accessed training (general/lipid) to support your clinical role; other information, skills and training that you may need to help you optimise lipid management;
- Any other questions or comments and suggestions that you want to mention on how you provide services for patients with CVD and what additional support you might need;
- Any other questions you have about this study.

Fu Y, *et al. BMJ Open* 2022; 12:e058951. doi: 10.1136/bmjopen-2021-058951
