# Supplementary material for: Tailoring lipid management interventions to reduce inequalities in cardiovascular disease risk management in primary care for deprived communities in Northern England: a mixed-methods intervention development protocol
Source: BMJ Open. 2022 Jul 4;12(7):e058951. doi: 10.1136/bmjopen-2021-058951 (PMC9255393; doi:10.1136/bmjopen-2021-058951)
Supplement: Author's manuscript [file bmjopen-2021-058951.draft_revisions.pdf]

BMJ Open

BMJ Open is committed to open peer review. As part of this commitment we make the peer review history of every article we publish publicly available.

When an article is published we post the peer reviewers' comments and the authors' responses online. We also post the versions of the paper that were used during peer review. These are the versions that the peer review comments apply to.

The versions of the paper that follow are the versions that were submitted during the peer review process. They are not the versions of record or the final published versions. They should not be cited or distributed as the published version of this manuscript.

BMJ Open is an open access journal and the full, final, typeset and author-corrected version of record of the manuscript is available on our site with no access controls, subscription charges or pay-per-view fees (http://bmjopen.bmj.com).

If you have any questions on BMJ Open's open peer review process please email info.bmjopen@bmj.com

BMJ Open

Tailoring lipid management interventions to reduce inequalities in cardiovascular risks and improve outcomes in deprived communities: intervention development protocol

Journal:	*BMJ Open*
Manuscript ID	bmjopen-2021-058951
Article Type:	Protocol
Date Submitted by the Author:	03-Nov-2021
Complete List of Authors:	Fu, Yu; Newcastle University Faculty of Medical Sciences, Population Health Sciences Institute<br>Tang, Eugene; Newcastle University Faculty of Medical Sciences<br>Sowden, Sarah; Newcastle University, Institute Health and Society;<br>Newton, Julia L.; Newcastle University<br>Whitty, Paula; Cumbria Northumberland Tyne and Wear NHS Foundation Trust
Keywords:	Cardiology < INTERNAL MEDICINE, PRIMARY CARE, PUBLIC HEALTH

SCHOLARONE™
Manuscripts

Tailoring lipid management interventions to reduce inequalities in cardiovascular risks and improve outcomes in deprived communities: intervention development protocol

Y Fu*[1], E Tang[2], S Sowden[2], J Newton[3], P Whitty[1]

1. National Institute for Health Research ARC North East and North Cumbria, Population Health Sciences Institute, Newcastle University, Newcastle, UK

2. Population Health Sciences Institute, Newcastle University, Newcastle, UK

3. Academic Health Science Network for the North East and North Cumbria, Newcastle, UK

*Correspondence

Dr Yu Fu

Baddiley-Clark Building

Richardson Road

Population Health Sciences Institute

Newcastle University

Newcastle

NE2 4AX

yu.fu@newcastle.ac.uk

01912 085307

Keywords: health inequalities; deprived populations; lipid management; mixed methods; protocol

Word count: 3500

For peer review only - http://bmjopen.bmj.com/site/about/guidelines.xhtml

ABSTRACT

Introduction

Hyperlipidaemia contributes a significant proportion of modifiable cardiovascular disease (CVD) risk, a condition that disproportionally affects disadvantaged socioeconomic communities, with death rates in the most deprived areas being four times higher than those in the least deprived. With the national CVD Prevention programme being delivered to minimise risk factors, no evidence is available on what has been implemented in primary care for deprived populations. This study describes the protocol for the development of a tailored intervention aiming to optimise lipid management in primary care settings to help reduce inequalities in CVD risks and improve outcomes in deprived communities.

Methods and analysis

A mixed methods approach will be employed consisting of four workpackages: 1) rapid review and logic model; 2) assessment of characteristics of lipid management; 3) interviews with health professionals; and 4) intervention development. A systematic search and narrative synthesis will be undertaken to identify evidence-based interventions and targeted outcomes in deprived areas. Practice level data will be assessed to establish the profile of lipid management, compared with the regional and national levels. Staff involved in the organisation and delivery of routine lipid management to deprived populations will be interviewed to understand the implementation and delivery of current lipid management and associated challenges. The prototype intervention will be informed by the evidence generated from workpackages 1-3, which will be reviewed and assessed using the nominal group technique to reach consensus. Training and skills development materials will also be developed as needed.

Ethics and dissemination

Ethics approval has been obtained from the Faculty of Medical Sciences Research Ethics Committee at Newcastle University, UK. Findings will be disseminated to the participating sites, participants, commissioners, and in peer-reviewed journals and academic conferences.

For peer review only - http://bmjopen.bmj.com/site/about/guidelines.xhtml

Strengths and limitations of this study

- This study will develop a tailored lipid management intervention for deprived populations to help reduce health inequalities, using multiple methods.
- Multiple data sources will be used to assess characteristics of lipid management in Deep End practices.
- Primary care staff needs and challenges in delivering current lipid management and resources related to implementation will be identified.
- Some limitations to the study design include exclusion of non-English studies, publication bias, quality of data and selection bias in the rapid evidence review.

For peer review only

INTRODUCTION

Hyperlipidaemia contributes a significant proportion of modifiable cardiovascular disease (CVD) risk, which is the leading cause of mortality and morbidity in England and Europe [1 2], accounting for a third of deaths in the UK. Hyperlipidaemia, a high level of cholesterol or triglycerides in the blood, can be inherited, is often found in people who are overweight, have alcohol abuse or have an unhealthy diet [3]. Elevated levels of blood lipids represent a major risk factor for the development of coronary heart disease (CHD) and other cerebrovascular diseases including stroke, transient ischaemic attack (TIA), and peripheral arterial disease. People with a history of these events are also at increased risk of experiencing subsequent CVDs. Management of CVDs also places a significant economic burden on the National Health Service (NHS), with an estimated cost of £7.4 billion per annum [4].

Both national and international guidelines recommend the use of statins in people at risk of CVD [1 5]. Their use aims to reduce the synthesis of cholesterol, but evidence suggests that there is an underuse of lipid-lowering drugs among eligible patients [6 7]. CVD is also a condition that is strongly associated with health inequalities and disproportionally affects disadvantaged socioeconomic communities. People in disadvantaged socioeconomic groups experience a higher prevalence of CVD events but poorer outcomes and premature mortality, leading to the fact that people in the most deprived areas in England are four times likely to die prematurely than those in the least deprived [8]. In addition, those living in socioeconomically disadvantaged neighbourhoods are found to have poor engagement with preventive health services, even though they are likely to benefit from screening and early treatment [9]. This may lead to an exacerbation of existing health inequalities. NICE guidance has recognised socioeconomic status as an additional factor that contributes to CVD risk. The NHS Long Term Plan [10] has also identified CVD as a clinical priority and stressed the wider impact on health inequalities, highlighting that heart disease-related mortality is the single largest contributor to the life expectancy gap between the most and least deprived. However, it failed to establish how health inequalities could be approached or addressed within local systems.

The North East of England is consistently ranked as having the highest poverty levels and the lowest health outcomes in England [11]. In early 2020, local GPs, Public Health leaders and academics collaborated to form a Deep End Steering Group for the region and funding was granted from the North East and North Cumbria Integrated Care System (NENC ICS) Prevention strand to establish and co-design a Deep End network for the region. The Deep End NENC network consists of 35 practices; practices identified as Deep End are those that fall into the 10% most deprived practice populations in England. This approach to definition mirrored that used in the Scottish Deep End project [12]. These practices have between 95.7% and 57.7% of registered patients living in the most deprived 15% of

For peer review only - http://bmjopen.bmj.com/site/about/guidelines.xhtml

indices of multiple deprivation (IMD) data zones. Due to the high rates of long term conditions, unhealthy diets and physical inactivity, together with other competing priorities [13], people in areas of deprivation are likely to face greater challenges in managing CVDs. Ongoing effects from the pandemic are exacerbating these challenges and include difficulties attending review appointments in person, digital poverty impeding remote review, low levels of health literacy resulting in misunderstandings about the need to continue long term treatments, and closure of other support services [14], potentially widening health inequalities.

The NHS has set up the national CVD Prevention programme [15] which aims to develop targeted interventions to minimise risk factors by maximising diagnosis and treatment, accompanied by the GP contract to commission a new national CVD prevention audit for primary care [16]. However, no evidence is available on what and how the intervention has been implemented and for what health outcomes for deprived populations. There is therefore an urgent need to seek a theoretical underpinning to tailor the national programme in this context, which could support the CVD element of the NHS post-COVID-19 recovery plan with the region.

This study will examine the literature and practice-level data and undertake engagement with staff who provide primary care for deprived populations to define the components and mechanisms through which lipid management can be optimised to meet the identified needs. The study aims to 1) synthesise the evidence on interventions for deprived populations with CVDs or those with high risks and understand the outcomes associated with these interventions, 2) establish the characteristics of current lipid management in primary care settings in deprived areas and identify clinical gaps and needs, 3) investigate the implementation and delivery of current interventions for patients with CVDs and those with high risks, and 4) tailor and optimise the national prevention programme to suit context and needs of deprived communities.

METHODS AND ANALYSIS

Study design

A mixed methods approach will be employed to inform the development of the intervention comprising a rapid review, an assessment of lipid management profile and qualitative interviews. Four work packages (WPs) are proposed (Figure 1).

[insert Figure 1]

For peer review only - http://bmjopen.bmj.com/site/about/guidelines.xhtml

A project advisory group consisting of 6-8 members will be established to involve key opinion leaders across core fields, who will advise at each project stage, review intervention components for the consensus process and help disseminate the study outputs. Members will recruit from the Deep End network, ICS Prevention Board, Academic Health Science Network (AHSN) NENC lipid steering group, regional professional leads for lipid management, public members and academics with methodology expertise. This group will meet quarterly with the research team to oversee the execution of the study and provide advice and assistance.

Patient and public involvement

The design of this study consulted the Deep End Steering group, which helped to shape the research focus and question. There was no direct involvement of patients. However, patients' experiences are central to the research question and outcomes, which has been recognised as an urgent agenda to aim to develop a patient and public involvement strategy. It will strengthen the conduct of this study and its dissemination.

WP1: Rapid review and logic model

WP 1 will be a rapid review and synthesis of current evidence, aiming to identify evidence-based interventions for lipid management in deprived areas and targeted outcomes. The review will be conducted following Cochrane guidance on rapid reviews [17]. A logic model will be developed, informed by existing literature to describe how lipid management works in theory to benefit services and patients.

Type of studies

Empirical studies (i.e. original data collection) describing the setting, problem addressed, resource requirements, aim, intervention components, provider, method of delivery and objective and subjective outcomes will be included if conducted in an Organisation for Economic Co-operation and Development (OECD) country [18] (to ensure a degree of commonality in health system and socioeconomic and demographic context), published in peer-reviewed scientific journals, within the last 10 years (to mirror the NHS long term plan) and in the English language.

Type of participants

Studies that focus on people with disadvantaged socioeconomic status (education, income, occupation, social class, deprivation, poverty or an area-based proxy for deprivation derived from place of residence) will be included. Adults with CVD including angina, previous myocardial infarction (MI), revascularisation, stroke or TIA or symptomatic peripheral arterial disease, and those who do not have established CVD but are identified as having a high risk of developing CVDs [1] considering age, ethnicity, socioeconomic status, BMI, history of taking antihypertensive or lipid modification

For peer review only - http://bmjopen.bmj.com/site/about/guidelines.xhtml

For peer review only

therapy, QRISK≥10%, diabetes, nephropathy, familial hypercholesterolaemia or other inherited disorder of lipid metabolism, and other underlying medical conditions or treatment including people treated for HIV, with serious mental health problems, taking medicines that can cause dyslipidaemia or with autoimmune disorders.

Type of interventions

Multifaceted interventions delivered to deprived populations that aim to optimise care by maximising diagnosis and/or treatment to minimise individual risk factors will be considered.

Type of outcome measures

Studies with individual, area-based or both types of measures of socioeconomic deprivation will be included. This may be measured according to several characteristics including income, employment, education, disability, crime, housing and services and living environment deprivation. Because there is no universal recommendation for core outcome sets in studies on CVD prevention [19-21], studies will be eligible for inclusion regardless of outcomes measured or reported for health outcomes, this may include vascular-related outcomes, cognitive and functional outcomes, lifestyle, medical risk factors, cardioprotective medications and patient-reported outcome measures. Any measures of professionals', patients' and/or families' knowledge, attitudes or satisfaction will also be included.

Study identification

Cochrane CENTRAL, MEDLINE, PsycInfo, CINAHL will be searched for eligible studies. Detailed search strategies will be developed for each database. A preliminary search strategy developed for MEDLINE is designed by YF and validated by an information specialist (online supplementary material 1).

Study selection

Identified citations will be exported to Endnote X9 [22] for deduplication and screening. A random selection (10%) of study titles and abstracts will be screened independently by another researcher. Full text will be retrieved where citations appeared to meet the eligibility criteria or where a decision to exclude will not be made on the information provided. Any discrepancies will be resolved by discussion with a third researcher.

Data extraction

Data will be extracted on author's first name, publication date, location (country in which the study was undertaken), study design, sample size, intervention details, control/comparison groups (if any), outcome measures and results, using a data extraction sheet that will be piloted on two retrieved study reports. Accuracy and consistency will be monitored through random double-extraction of

For peer review only - http://bmjopen.bmj.com/site/about/guidelines.xhtml

10% included studies by an independent researcher. Any discrepancies will be resolved by discussion with a third researcher. Where a study appears to have multiple citations, original authors will be contacted for clarification. All information from multiple citations will be used if no replies received.

Quality assessment

Quality appraisal of included studies will be performed using standardised tools adapted for purpose. Appropriate Critical Appraisal Skills Programme (CASP) tool will be used according to the study design, a random sample (10%) will be independently assessed by another researcher. Any discrepancies will be resolved by discussion with a third researcher.

Data synthesis

A narrative synthesis will be undertaken following Popay et al's [23] approach to conducting synthesis systematically and transparently. It will focus on the intervention components, effects of the interventions and mechanisms leading to the outcomes. Studies, interventions and outcomes will be examined and grouped according to the aim and components of the interventions. The variation on different characteristics of health systems will be taken into account when interpreting the intervention across OECD countries. A logic model will be produced to present context, intervention components and outcomes. Possible unintended adverse outcomes will also be provided.

WP2: Assessment of characteristics of lipid management

WP2 will be a quantitative assessment of characteristics of lipid management in Deep End practices using publicly accessible datasets and anonymised data requested from the North East Commissioning Support Unit (NECSU).

Data sources

The primary data source for this study will be the GP Practice Profiles and CVD Profiles from Public Health Profiles[24] via Fingertips, a publicly accessible web tool containing national general practice profiles generated for all Quality and Outcomes Framework (QOF)[25] 2019/20 with a list size of at least 750 patients. Available practice-level data include local demography, QOF domains and patient satisfaction. Fingertips also provides access to CVD profiles for each clinical commissioning group (CCG) that include data on mortality, hospital admissions, procedures and disease management. Other data sources used include the QOF, OpenPerscribing[26] and data requested from the NECSU via electronic Prescribing Analysis and Costs (ePACT2)[27] and Secondary Uses Services (SUS)[28] data (Table 1).

For peer review only - http://bmjopen.bmj.com/site/about/guidelines.xhtml

Table 1 Data sources and variables

Data source	Description	Level of data available	Variables and variable description
GP Practice Profiles	Date reported by GPs to the NHS that refers to all patients in a practice	• Individual practice	• Practice size<br>• Mean practice age<br>• Deprivation score<br>• Age groups<br>• Percentage of patients positive experiences as “good”<br>• Percentage of practice access rated by patients as “good”<br>• Percentage with a long term condition<br>• Education status<br>• Working status<br>• Life expectancy by sex
Cardiovascular disease profiles	Overview of data on CVD and CVD related conditions of heart disease and stroke, including data on mortality, hospital	• Individual practice	• Estimated smoking prevalence<br>• Record of offer of support and treatment for smoking<br>• Patients on the CHD register with a record that aspirin, alternative anti-platelet therapy (APT), or an anticoagulant (ACT) is taken<br>• Percentage of patients with heart failure due to left ventricular dysfunction (LVD) who are treated with ACE inhibitor or angiotensin receptor blocker (ARB)<br>• Percentage of patients with a stroke who have a record that APT or an ACT is taken<br>• Percentage of patients with atrial fibrillation (if CHADS2DS2-VASc >=2) treated with ACT
QOF	An indication of the overall achievement of a practice through a points system, concerning clinical, public health, public health – additional services, and quality improvement	• Individual practice	• Total on the atrial fibrillation (AF) register, prevalence<br>• Total on the CVD-primary prevention (CVD-PP) register, prevalence<br>• Total on the CHD register, prevalence<br>• Total on the heart failure (HF) register, prevalence<br>• Total on the left ventricular systolic dysfunction (LVSD) register, prevalence

For peer review only - http://bmjopen.bmj.com/site/about/guidelines.xhtml

			• Total on the hypertension (HYP) register, prevalence<br>• Total on the peripheral arterial disease (PAD) register, prevalence<br>• Total on the stroke and transient ischaemic attack (STIA) register, prevalence<br>• QOF score
OpenPrescribing	Imports national prescribing data published by NHS Business Services Authority	• Individual practice<br>• CCG level	• Total statin<br>• Total low and medium intensity statin
ePACT2	An online business intelligence tool to view analyse and present prescribing data. The data was aggregated at the CCG level monthly.	• Individual practice	• Total taking atorvastatin<br>• Total taking fluvastatin<br>• Total taking pravastatin<br>• Total taking rosuvastatin<br>• Total taking simvastatin<br>• Total taking ezetimibe<br>• Total taking simvastatin and ezetimibe<br>• Statins (BNF 2.12 sub-set) ADQ/STAR-PU (Average Daily Quantity/Specific Therapeutic group Age-sex Related Prescribing Unit)
SUS, NHS Digital	It provides right time access to A&E. aggregate level for benchmarking and trend analysis.	• Individual practice	• A&E Attendances where first or second A&E Emergency Diagnosis code is cardiac condition

Study population

The study population are patients aged 16 and above who have been diagnosed with any form of CVDs recorded in Office for Health Improvement and Disparities database data from 2019 to 2020, which is the study period of interest. The dataset is aggregated to the GP practice level in which variables are summarised if they are at the patient level. The data linkage will be performed by using the unique GP practice code.

Data analysis

Descriptive analysis will be used to provide a quick and low cost approach to describe characteristics of lipid management and give descriptive statistics or investigate relationships between factors. Continuous variables will be summarised using means and standard deviations or medians and interquartile ranges, while percentages will be used to summarise binary variables. Statistics will be presented at practice, clinical commissioning group (CCG) and national (England) level. Comparisons

For peer review only

For peer review only - http://bmjopen.bmj.com/site/about/guidelines.xhtml

will be made between practice and CCG (which practices belong) level. The t-test (or Mann-Whitney U test) will be used to compare continuous variables and the x^2 test (or Fisher's exact test) will be used for similar comparisons of categorical variables. Confidence intervals for differences in means, medians or percentages will be calculated. All significance tests will be performed at the 5% level. Stata version 16 will be used to facilitate data analysis.

WP3: Interviews with health professionals

WP3 will be qualitative interviews with staff involved in the organisation and delivery of routine lipid management in practices that are part of the Deep End Network. The aim of the interviews will be to understand the implementation and delivery of current lipid management and identify their needs and challenges.

Participants and recruitment

Staff involved in the organisation and delivery of routine lipid management in practices are eligible to participate in this study. Pharmacists and social prescribers in general practice also have a key role in supporting the management of dyslipidaemia, therefore consulting clinician and the facilitator a healthcare assistant would also be invited.

Study recruitment will be supported by the Deep End practice Network, who will send an email containing brief study information to healthcare staff working in participating practices. Staff can express their interests by responding to the email straight to the research team. A reminder will be sent to those who have not responded in two weeks. Maximum variation sampling will be used to ensure broad representation of staff on dimensions including job titles/roles, grade, speciality, length of working, COVID-19 exposure, and demographics. Reasons given by practices for declining to participate will be recorded to inform feasibility assessment to further studies.

Data collection

With participants' informed written consent, semi-structured interviews will be conducted via telephone or online (i.e. Zoom) for up to 60 mins. A topic guide that has already been developed will be piloted with a couple of health professionals prior to data collection and will be continuously updated in line with emerging themes and participants' feedback. Data collection will end when data saturation is reached indicating no new information is discovered; we anticipate approximately 20-25 interviews.

Data analysis

Interviews will be digitally recorded (with consent), transcribed and data managed using NVivo 12, a qualitative software programme to assist with the organisation and coding of data. Data will be

For peer review only - http://bmjopen.bmj.com/site/about/guidelines.xhtml

analysed using Framework Analysis, which provides a systematic approach to sifting, charting, and sorting material using the key themes and issues. To ensure trustworthiness and rigour of the analysis, the coding framework will be developed and assured by double coding of a random sample of transcripts (10%) as a validity check and exploring alternative interpretations of the data.

WP4: Intervention development

WP 4 will develop the prototype of the intervention in collaboration with the Academic Health Science Network NENC which delivers the CVD Prevention programme, part of which includes a national programme mandated by NHS England and NHS Improvement.

Guided by the MRC Framework for developing and evaluating complex interventions [29], the development of intervention will be informed by integrating the outcomes of the literature evidence, current CVD management profile, and stakeholder engagement undertaken in WPs1-3, in an iterative and progressive approach. The national programme and its key components will be examined against the gaps, needs, and challenges identified to consider the wider context. The prototype intervention will be designed taking account of health professionals' existing commitments in these practices and challenging working environments. Training and skills development materials for staff will also be developed to facilitate them in delivering the tailored intervention. The logic model produced in WP1 will be refined to map key intervention processes and outcomes.

Design

The prototype intervention will be reviewed and assessed by the project advisory group, guided by a nominal group technique, a consensus method that allows for the generation of views and thoughts from group participants whilst maintaining anonymity throughout [30].

Data collection

The group will be provided with details of the interventions and refined logic model, to seek further comments and explore if the intervention is feasible, acceptable, and implementable in the context. The APEASE criteria [31] will be used to determine the acceptability, practicability, effectiveness, affordability, side-effects, and equity aspects of the intervention. The nominal group technique will involve two main sections:

1. The group will be asked to provide their comments on the intervention, training materials, and logic model. All comments will be collated and grouped into main themes for each member to rate their top 10 priorities of the comments. Group ratings will be summated, and the group's collective top 10 priorities will be presented to the group and discussed.

For peer review only - http://bmjopen.bmj.com/site/about/guidelines.xhtml

2. Each will re-rate the group's top 10 priorities and provide a weighting for their top 10 comments in the scale ranging from 1=least important to 100=most important. These weightings will be summated after the meeting, which will be used to refine the intervention and the logic model. The refined version will be sent out to each member for further comments. It is expected that this process will be repeated twice until a census is reached.

Data analysis

The initial listing of comments, clarification, and discussion of comments in section 1 will be analysed thematically, with further discussion with the research team. The scale data generated in section 2 will be averaged so that the comments can be re-ordered according to weighted ranked priority. The individual/group rankings produced in sections 1 and 2 will be compared to estimate the level of agreement between the sections and to observe the process of reaching consensus. Firstly, these comparisons will be made by calculating the percentage agreement between the sections, in terms of the comments that appear in the top ten priorities each time. Secondly, the movement in ranking between sections 1 and 2 will be estimated using Cohen's kappa statistic of chance-corrected agreement [32]. A kappa value of >0.40 is considered to represent a moderate level of agreement [33].

ETHICS AND DISSEMINATION

Ethics approval has been sought from the Faculty of Medical Sciences Research Ethics Committee at Newcastle University (reference no.: 2209/14251) UK, a letter of support has also been issued by the NENC Deep End Network.

Dissemination will be led by the research team and supported by the project advisory group. Reports will be produced and shared with the NENC ICS Prevention Board, Inequalities Board, Deep End network, National Institute for Health Research (NIHR) Applied Research Collaboration (ARC) NENC, and AHSN NENC. The findings will be disseminated to the participating sites, participants, commissioners, and in peer-reviewed journals and academic conferences.

For peer review only

For peer review only - http://bmjopen.bmj.com/site/about/guidelines.xhtml

FIGURES

Figure 1 Study design and related work packages

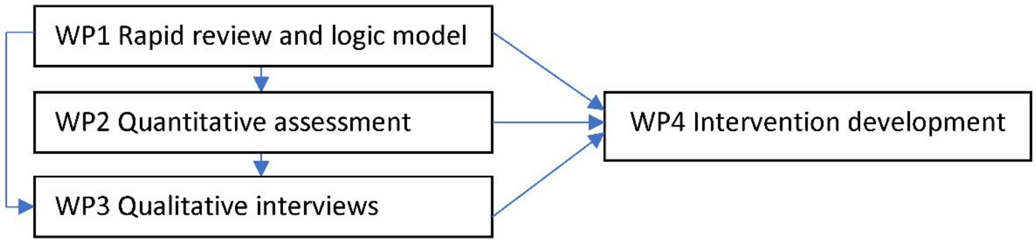

For peer review only

For peer review only - http://bmjopen.bmj.com/site/about/guidelines.xhtml

ACKNOWLEDGEMENT

We thank NENC Deep End Steering Group and NECSU for their support in this study.

AUTHORS' CONTRIBUTIONS

YF, JN and PW conceived the study design. YF drafted and revised the manuscript, and obtained the ethics approval. ET, SS, JN and PW independently reviewed and contributed to revising and approving the final version.

FUNDING

This project/research/work is supported by the National Institute of Health Research (NIHR) Applied Research Collaboration (ARC) for the North East and North Cumbria (NENC) (grant number not applicable). The NIHR is the nation's largest funder of health and care research and provides the people, facilities and technology that enables research to thrive. Find out more about the NIHR.

COMPETING INTERESTS

None declared.

For peer review only

For peer review only - http://bmjopen.bmj.com/site/about/guidelines.xhtml

REFERENCES

1. NICE. Cardiovascular disease: risk assessment and reduction, including lipid modification National Institute for Health and Care Excellence; 2014 [accessed 19th July 2021.
2. Eurostat. Cardiovascular diseases statistics: Eurostat; 2020 [Available from: https://ec.europa.eu/eurostat/statistics-explained/index.php?title=Cardiovascular_diseases_statistics#Deaths_from_cardiovascular_diseases accessed 20th July 2021.
3. Nelson RH. Hyperlipidemia as a risk factor for cardiovascular disease. *Prim Care* 2013;40(1):195-211. doi: 10.1016/j.pop.2012.11.003 [published Online First: 2012/12/04]
4. Public Health England. Health matters: preventing cardiovascular disease: Public Health England; 2019 [Available from: https://www.gov.uk/government/publications/health-matters-preventing-cardiovascular-disease/health-matters-preventing-cardiovascular-disease accessed 20th July 2021.
5. Mach F, Baigent C, Catapano AL, et al. 2019 ESC/EAS Guidelines for the management of dyslipidaemias: lipid modification to reduce cardiovascular risk: The Task Force for the management of dyslipidaemias of the European Society of Cardiology (ESC) and European Atherosclerosis Society (EAS). *European Heart Journal* 2019;41(1):111-88. doi: 10.1093/eurheartj/ehz455
6. Wu J, Zhu S, Yao GL, et al. Patient Factors Influencing the Prescribing of Lipid Lowering Drugs for Primary Prevention of Cardiovascular Disease in UK General Practice: A National Retrospective Cohort Study. *PLOS ONE* 2013;8(7):e67611. doi: 10.1371/journal.pone.0067611
7. Ray KK, Molemans B, Schoonen WM, et al. EU-Wide Cross-Sectional Observational Study of Lipid-Modifying Therapy Use in Secondary and Primary Care: the DA VINCI study. *European Journal of Preventive Cardiology* 2020 doi: 10.1093/eurjpc/zwaa047
8. NHS. Optimising treatment of high-risk conditions: NHS; 2021 [Available from: https://www.england.nhs.uk/ltphimenu/cvd/optimising-treatment-of-high-risk-conditions/ accessed 28th September 2021.
9. Lang S-J, Abel GA, Mant J, et al. Impact of socioeconomic deprivation on screening for cardiovascular disease risk in a primary prevention population: a cross-sectional study. *BMJ Open* 2016;6(3):e009984. doi: 10.1136/bmjopen-2015-009984
10. NHS. The NHS Long Term Plan London: National Health Service; 2019 [Available from: https://www.longtermplan.nhs.uk/wp-content/uploads/2019/08/nhs-long-term-plan-version-1.2.pdf accessed 20th July 2021.
11. Corris V, Dormer E, Brown A, et al. Health inequalities are worsening in the North East of England. *British Medical Bulletin* 2020;134(1):63-72. doi: 10.1093/bmb/ldaa008
12. Watt G, Brown G, Budd J, et al. General Practitioners at the Deep End: The experience and views of general practitioners working in the most severely deprived areas of Scotland. *Occasional paper (Royal College of General Practitioners)* 2012(89):i.
13. Holmes J. Tackling obesity: the role of the NHS in a whole-system approach: The King's Fund; 2021 [Available from: https://www.kingsfund.org.uk/sites/default/files/2021-07/Tackling%20obesity.pdf accessed 20th July 2021.
14. Norman C, Wildman JM, Sowden S. COVID-19 at the Deep End: A Qualitative Interview Study of Primary Care Staff Working in the Most Deprived Areas of England during the COVID-19 Pandemic. *International Journal of Environmental Research and Public Health* 2021;18(16):8689.
15. NHS England. Cardiovascular disease London: National Health Service,; 2019 [Available from: https://www.england.nhs.uk/ourwork/clinical-policy/cvd/ accessed 20th July 2021.
16. NHS England. CVDPREVENT London: National Health Service; 2019 [Available from: https://www.england.nhs.uk/ourwork/clinical-policy/cvd/cvdprevent/ accessed 20th July 2021.

For peer review only - http://bmjopen.bmj.com/site/about/guidelines.xhtml

17. Garritty C, Gartlehner G, Kamel C, et al. Cochrane Rapid Reviews Interim Guidance from the Cochrane Rapid Reviews Methods Group. March 2020, 2020.
18. Reibling N, Ariaans M, Wendt C. Worlds of Healthcare: A Healthcare System Typology of OECD Countries. *Health Policy* 2019;123(7):611-20. doi: https://doi.org/10.1016/j.healthpol.2019.05.001
19. McNamara RL, Spatz ES, Kelley TA, et al. Standardized Outcome Measurement for Patients With Coronary Artery Disease: Consensus From the International Consortium for Health Outcomes Measurement (ICHOM). *Journal of the American Heart Association* 2015;4(5):e001767. doi: doi:10.1161/JAHA.115.001767
20. Neilson LM, Swart ECS, Good CB, et al. Identifying Outcome Measures for Coronary Artery Disease Value-Based Contracting Using the Delphi Method. *Cardiology and Therapy* 2019;8(1):135-43. doi: 10.1007/s40119-019-0132-7
21. Canavan M, Smyth A, Robinson SM, et al. Attitudes to outcomes measured in clinical trials of cardiovascular prevention. *QJM: An International Journal of Medicine* 2015;109(6):391-97. doi: 10.1093/qjmed/hcv132
22. Hupe M. EndNote X9. *Journal of Electronic Resources in Medical Libraries* 2019;16(3-4):117-19. doi: 10.1080/15424065.2019.1691963
23. Popay J, Roberts H, Sowden A, et al. Guidance on the conduct of narrative synthesis in systematic reviews. *A product from the ESRC methods programme Version* 2006;1:b92.
24. Public Health England. Public Health Profiles.: Public Health England; 2021 [Available from: https://fingertips.phe.org.uk accessed 29th Oct 2021.
25. NHS Digital. Quality Outcomes Framework (QOF): NHS Digital; 2021 [accessed 29th October 2021.
26. OpenPrescribing.net. EBM DataLab, University of Oxford, 2020.
27. NHS Business Services Authority. ePACT2: NHS Business Services Authority; 2021 [Available from: https://www.nhsbsa.nhs.uk/access-our-data-products/epact2 accessed 29th Oct 2021.
28. NHS Digital. Secondary Uses Service (SUS): NHS Digital; 2021 [Available from: https://digital.nhs.uk/services/secondary-uses-service-sus accessed 29th Oct 2021.
29. Craig P, Dieppe P, Macintyre S, et al. Developing and evaluating complex interventions: the new Medical Research Council guidance. *Bmj* 2008;337
30. Harvey N, Holmes CA. Nominal group technique: an effective method for obtaining group consensus. *International journal of nursing practice* 2012;18(2):188-94.
31. West R, Michie S, Atkins L, et al. Achieving behaviour change: A guide for local government and partners London: Public Health England; 2019 [Available from: https://assets.publishing.service.gov.uk/government/uploads/system/uploads/attachment_data/file/875385/PHEBI_Achieving_Behaviour_Change_Local_Government.pdf accessed 14th September 2021.
32. Cohen J. A coefficient of agreement for nominal scales. *Educational and Psychological Measurement* 1960
33. Landis JR, Koch GG. The measurement of observer agreement for categorical data. *biometrics* 1977:159-74.

For peer review only

For peer review only - http://bmjopen.bmj.com/site/about/guidelines.xhtml

Supplementary Material 1 Search strategy for MEDLINE (Ovid) (1996 to October Week 4 2021)

1. exp Cardiovascular Diseases/
2. cardio*.tw.
3. cardia*.tw.
4. heart*.tw.
5. coronary*.tw.
6. angina*.tw.
7. ventric*.tw.
8. myocard*.tw.
9. pericard*.tw.
10. isch?em*.tw.
11. emboli*.tw.
12. arrhythmi*.tw.
13. thrombo*.tw.
14. atrial fibrillat*.tw.
15. tachycardi*.tw.
16. endocardi*.tw.
17. (sick adj sinus).tw.
18. exp Stroke/

For peer review only - http://bmjopen.bmj.com/site/about/guidelines.xhtml

19. (stroke or strokes).tw.
20. cerebrovasc*.tw.
21. cerebral vascular.tw.
22. apoplexy.tw.
23. ((brain* or cerebral or lacunar) adj2 infarct*).tw.
24. exp Hypertension/
25. hypertensi*.tw.
26. peripheral arter* disease*.tw.
27. ((high or increased or elevated) adj2 blood pressure).tw.
28. exp Hyperlipidemias/
29. hyperlipid*.tw.
30. hyperlip?emia*.tw.
31. hypercholesterol*.tw.
32. hypercholester?emia*.tw.
33. hyperlipoprotein?emia*.tw.
34. hypertriglycerid?emia*.tw.
35. exp Arteriosclerosis/
36. exp Cholesterol/
37. cholesterol.tw.
38. Blood Pressure/

For peer review only - http://bmjopen.bmj.com/site/about/guidelines.xhtml

For peer review only

39. 1 or 2 or 3 or 4 or 5 or 6 or 7 or 8 or 9 or 10 or 11 or 12 or 13 or 14 or 15 or 16 or 17 or 18 or 19 or 20 or 21 or 22 or 23 or 24 or 25 or 26 or 27 or 28 or 29 or 30 or 31 or 32 or 33 or 34 or 35 or 36 or 37 or 38
40. exp Socioeconomic Factors/
41. exp social class/
42. socioecon*.tw.
43. demographic*.tw.
44. disadvantage*.tw.
45. disparit*.tw.
46. deprivation.tw.
47. exp Health Services Accessibility/
48. deprive*.tw.
49. pover*.tw.
50. inequalit*.tw.
51. education*.tw.
52. unemploy*.tw.
53. employed.tw.
54. employment.tw.
55. income.tw.
56. occupation*.tw.
57. SES.tw.

For peer review only

58. class.tw.

59. economic.tw.

60. (social adj1 (class or factor or factors)).tw.

61. 40 or 41 or 42 or 43 or 44 or 45 or 46 or 47 or 48 or 49 or 50 or 51 or 52 or 53 or 54 or 55 or 56 or 57 or 58 or 59 or 60

62. exp General Practitioners/

63. exp General Practice/

64. general pract*.tw.

65. exp Family Practice/

66. family pract*.tw.

67. family medicine/

68. exp Family Practice/

69. exp Physicians, Family/

70. family phys*.tw.

71. exp Nurse Practitioners/

72. exp Nurse Clinicians/

73. nurse pract*.tw.

74. nurse clinic*.tw.

75. exp Primary Health Care/

76. primary care.tw.

77. exp Community Health Services/

For peer review only - http://bmjopen.bmj.com/site/about/guidelines.xhtml

For peer review only

78. community care.tw.

79. 62 or 63 or 64 or 65 or 66 or 67 or 68 or 69 or 70 or 71 or 72 or 73 or 74 or 75 or 76 or 77 or 78

80. 39 and 61 and 79

81. (Algeria$ or Egypt$ or Liby$ or Morocc$ or Tunisia$ or Western Sahara$ or Angola$ or Benin or Botswana$ or Burkina Faso or Burundi or Cameroon or Cape Verde or Central African Republic or Chad or Comoros or Congo or Djibouti or Eritrea or Ethiopia$ or Gabon or Gambia$ or Ghana or Guinea or Keny$ or Lesotho or Liberia or Madagasca$ or Malawi or Mali or Mauritania or Mauritius or Mayotte or Mozambiq$ or Namibia$ or Niger or Nigeria$ or Reunion or Rwand$ or Saint Helena or Senegal or Seychelles or Sierra Leone or Somalia or South Africa$ or Sudan or Swaziland or Tanzania or Togo or Ugand$ or Zambia$ or Zimbabw$ or China or Chinese or Hong Kong or Macao or Mongolia$ or Taiwan$ or Belarus or Moldov$ or Russia$ or Ukraine or Afghanistan or Armenia$ or Azerbaijan or Bahrain or Cyprus or Cypriot or Georgia$ or Iran$ or Iraq$ or Israel$ or Jordan$ or Kazakhstan or Kuwait or Kyrgyzstan or Leban$ or Oman or Pakistan$ or Palestin$ or Qatar or Saudi Arabia or Syria$ or Tajikistan or Turkmenistan or United Arab Emirates or Uzbekistan or Yemen or Bangladesh$ or Bhutan or British Indian Ocean Territory or Brunei Darussalam or Cambodia$ or India$ or Indonesia$ or Lao or People's Democratic Republic or Malaysia$ or Maldives or Myanmar or Nepal or Philippin$ or Singapore or Sri Lanka or Thai$ or Timor Leste or Vietnam or Albania$ or Andorra or Bosnia$ or Herzegovina$ or Bulgaria$ or Croatia$ or Estonia or Faroe Islands or Greenland or Liechtenstein or Lithuani$ or Macedonia or Malta or maltese or Romania or Serbia$ or Montenegro or Slovenia or Svalbard or Argentina$ or Belize or Bolivia$ or Brazil$ or chile or Chilean or Colombia$ or Costa Rica$ or Cuba or Ecuador or El Salvador or French Guiana or Guatemala$ or Guyana or Haiti or Honduras or Jamaica$ or Nicaragua$ or Panama or Paraguay or Peru or Puerto Rico or Suriname or Uruguay or Venezuela or developing countr$ or south America$).ti,sh. [mp=title, abstract, original title, name of substance word, subject heading word, floating sub-heading word, keyword heading word, organism supplementary concept word, protocol supplementary concept word, rare disease supplementary concept word, unique identifier, synonyms]

82. 80 not 81

83. limit 82 to (abstracts and english language and humans and yr="2011 -Current")

For peer review only

PRISMA-P (Preferred Reporting Items for Systematic review and Meta-Analysis Protocols) 2015 checklist: recommended items to address in a systematic review protocol*

Section and topic	Item No	Checklist item	Page no.
**ADMINISTRATIVE INFORMATION**			
Title:			
Identification	1a	Identify the report as a protocol of a systematic review	
Update	1b	If the protocol is for an update of a previous systematic review, identify as such	
Registration	2	If registered, provide the name of the registry (such as PROSPERO) and registration number	
Authors:			
Contact	3a	Provide name, institutional affiliation, e-mail address of all protocol authors; provide physical mailing address of corresponding author	
Contributions	3b	Describe contributions of protocol authors and identify the guarantor of the review	
Amendments	4	If the protocol represents an amendment of a previously completed or published protocol, identify as such and list changes; otherwise, state plan for documenting important protocol amendments	
Support:			
Sources	5a	Indicate sources of financial or other support for the review	
Sponsor	5b	Provide name for the review funder and/or sponsor	
Role of sponsor or funder	5c	Describe roles of funder(s), sponsor(s), and/or institution(s), if any, in developing the protocol	
**INTRODUCTION**			
Rationale	6	Describe the rationale for the review in the context of what is already known	5-6
Objectives	7	Provide an explicit statement of the question(s) the review will address with reference to participants, interventions, comparators, and outcomes (PICO)	6
**METHODS**			
Eligibility criteria	8	Specify the study characteristics (such as PICO, study design, setting, time frame) and report characteristics (such as years considered, language, publication status) to be used as criteria for eligibility for the review	7-8
Information sources	9	Describe all intended information sources (such as electronic databases, contact with study authors, trial registers or other grey literature sources) with planned dates of coverage	8
Search strategy	10	Present draft of search strategy to be used for at least one electronic database, including planned limits, such that it could be	Supplementary

For peer review only

		repeated	
Study records:			
Data management	11a	Describe the mechanism(s) that will be used to manage records and data throughout the review	8
Selection process	11b	State the process that will be used for selecting studies (such as two independent reviewers) through each phase of the review (that is, screening, eligibility and inclusion in meta-analysis)	8
Data collection process	11c	Describe planned method of extracting data from reports (such as piloting forms, done independently, in duplicate), any processes for obtaining and confirming data from investigators	8
Data items	12	List and define all variables for which data will be sought (such as PICO items, funding sources), any pre-planned data assumptions and simplifications	9
Outcomes and prioritization	13	List and define all outcomes for which data will be sought, including prioritization of main and additional outcomes, with rationale	9
Risk of bias in individual studies	14	Describe anticipated methods for assessing risk of bias of individual studies, including whether this will be done at the outcome or study level, or both; state how this information will be used in data synthesis	8-9
Data synthesis	15a	Describe criteria under which study data will be quantitatively synthesised	9
	15b	If data are appropriate for quantitative synthesis, describe planned summary measures, methods of handling data and methods of combining data from studies, including any planned exploration of consistency (such as I^2, Kendall's τ)	
	15c	Describe any proposed additional analyses (such as sensitivity or subgroup analyses, meta-regression)	
	15d	If quantitative synthesis is not appropriate, describe the type of summary planned	
Meta-bias(es)	16	Specify any planned assessment of meta-bias(es) (such as publication bias across studies, selective reporting within studies)	n/a
Confidence in cumulative evidence	17	Describe how the strength of the body of evidence will be assessed (such as GRADE)	n/a

*** It is strongly recommended that this checklist be read in conjunction with the PRISMA-P Explanation and Elaboration (cite when available) for important clarification on the items. Amendments to a review protocol should be tracked and dated.** **The copyright for PRISMA-P (including checklist) is held by the PRISMA-P Group and is distributed under a Creative Commons Attribution Licence 4.0.**

From: Shamseer L, Moher D, Clarke M, Ghersi D, Liberati A, Petticrew M, Shekelle P, Stewart L, PRISMA-P Group. Preferred reporting items for systematic review and meta-analysis protocols (PRISMA-P) 2015: elaboration and explanation. BMJ. 2015 Jan 2;349(jan02 1):g7647.

For peer review only

BMJ Open

Tailoring lipid management interventions to reduce inequalities in cardiovascular disease risk management in primary care for deprived communities in Northern England: a mixed methods intervention development protocol

Journal:	*BMJ Open*
Manuscript ID	bmjopen-2021-058951.R1
Article Type:	Protocol
Date Submitted by the Author:	08-Apr-2022
Complete List of Authors:	Fu, Yu; Newcastle University Faculty of Medical Sciences, Population Health Sciences Institute<br>Tang, Eugene; Newcastle University Faculty of Medical Sciences<br>Sowden, Sarah; Newcastle University, Institute Health and Society;<br>Newton, Julia L.; Newcastle University<br>Whitty, Paula; Cumbria Northumberland Tyne and Wear NHS Foundation Trust
**Primary Subject Heading**:	General practice / Family practice
Secondary Subject Heading:	General practice / Family practice, Public health
Keywords:	Cardiology < INTERNAL MEDICINE, PRIMARY CARE, PUBLIC HEALTH

SCHOLARONE™
Manuscripts

Tailoring lipid management interventions to reduce inequalities in cardiovascular disease risk management in primary care for deprived communities in Northern England: a mixed methods intervention development protocol

Y Fu*[1,2], EYH Tang[2], S Sowden[2], J Newton[2,3], P Whitty[1]

1. National Institute for Health Research ARC North East and North Cumbria, Population Health Sciences Institute, Newcastle University, Newcastle, UK

2. Population Health Sciences Institute, Newcastle University, Newcastle, UK

3. Academic Health Science Network for the North East and North Cumbria, Newcastle, UK

*Correspondence

Dr Yu Fu

Baddiley-Clark Building

Richardson Road

Population Health Sciences Institute

Newcastle University

Newcastle

NE2 4AX

yu.fu@newcastle.ac.uk

01912 085307

Keywords: health inequalities; deprived populations; lipid management; mixed methods; protocol

Word count: 3500

For peer review only

ABSTRACT

Introduction

Hyperlipidaemia contributes a significant proportion of modifiable cardiovascular disease (CVD) risk, which is a condition that disproportionally affects disadvantaged socioeconomic communities, with death rates in the most deprived areas being four times higher than those in the least deprived. With the national CVD Prevention programme being delivered to minimise risk factors, no evidence is available on what has been implemented in primary care for deprived populations. This study describes the protocol for the development of a tailored intervention aiming to optimise lipid management in primary care settings to help reduce inequalities in CVD risks and improve outcomes in deprived communities.

Methods and analysis

A mixed methods approach will be employed consisting of four workpackages: 1) rapid review and logic model; 2) assessment and comparison of CVD risk management for deprived with non-deprived populations in Northern England to England overall; 3) interviews with health professionals; 4) intervention development. A systematic search and narrative synthesis will be undertaken to identify evidence-based interventions and targeted outcomes in deprived areas. General practice level data will be assessed to establish the profile of lipid management, compared with the regional and national levels. Health professionals involved in the organisation and delivery of routine lipid management to deprived populations will be interviewed to understand the implementation and delivery of current lipid management and associated challenges. The prototype intervention will be informed by the evidence generated from workpackages 1-3, which will be reviewed and assessed using the nominal group technique to reach consensus. Training and skills development materials will also be developed as needed.

Ethics and dissemination

Ethics approval has been obtained from the Faculty of Medical Sciences Research Ethics Committee at Newcastle University, UK. Findings will be disseminated to the participating sites, participants, commissioners, and in peer-reviewed journals and academic conferences.

For peer review only - http://bmjopen.bmj.com/site/about/guidelines.xhtml

Strengths and limitations of this study

- This study will develop a tailored lipid management intervention for deprived populations to help reduce health inequalities, using multiple methods.
- Multiple data sources will be used to assess and compare CVD risk management for deprived with non-deprived populations in Northern England to England overall.
- Primary care staff needs and challenges in delivering current lipid management and resources related to implementation will be identified.
- Some limitations to the study design include exclusion of non-English studies, publication bias, quality of data and selection bias in the rapid evidence review.

For peer review only

For peer review only - http://bmjopen.bmj.com/site/about/guidelines.xhtml

INTRODUCTION

Hyperlipidaemia contributes a significant proportion of modifiable cardiovascular disease (CVD) risk, which is the leading cause of mortality and morbidity in England and Europe [1 2], accounting for a third of deaths in the UK. Hyperlipidaemia, a high level of cholesterol or triglycerides in the blood, can be inherited, is often found in people who are overweight, have alcohol abuse or have an unhealthy diet [3]. Elevated levels of blood lipids represent a major risk factor for the development of coronary heart disease (CHD) and other cerebrovascular diseases including stroke, transient ischaemic attack (TIA), and peripheral arterial disease. People with a history of these events are also at increased risk of experiencing subsequent CVDs. Management of CVDs also places a significant economic burden on the National Health Service (NHS), with an estimated cost of £7.4 billion per annum [4].

Both national and international guidelines recommend the use of statins in people at risk of CVD [1 5]. Their use aims to reduce the synthesis of cholesterol, but evidence suggests that there is an underuse of lipid-lowering drugs among eligible patients [6 7]. CVD is also a condition that is strongly associated with health inequalities and disproportionally affects disadvantaged socioeconomic communities. People in disadvantaged socioeconomic groups experience a higher prevalence of CVD events but poorer outcomes and premature mortality, leading to the fact that people in the most deprived areas in England are four times more likely to die prematurely than those in the least deprived [8]. In addition, those living in socioeconomically disadvantaged neighbourhoods are found to have poor engagement with preventive health services, even though they are likely to benefit from screening and early treatment [9]. This may lead to an exacerbation of existing health inequalities. NICE guidance has recognised socioeconomic status as an additional factor that contributes to CVD risk. The NHS Long Term Plan [10] has also identified CVD as a clinical priority and stressed the wider impact on health inequalities, highlighting that heart disease-related mortality is the single largest contributor to the life expectancy gap between the most and least deprived. However, it failed to establish how health inequalities could be approached or addressed within local systems.

The North East of England is consistently ranked as having the highest poverty levels and the lowest health outcomes in England [11]. Scotland has established a programme to support general practices caring for the most deprived communities (the 'Deep End' project)[12] and, in early 2020, local GPs, Public Health leaders and academics collaborated to form a Deep End Steering Group for the North East and North Cumbria (NENC). Funding was then granted from the North East and North Cumbria Integrated Care System (NENC ICS) Prevention strand to establish and co-design a Deep End network for the region. The Deep End NENC network consists of 35 practices; practices identified as Deep End

For peer review only - http://bmjopen.bmj.com/site/about/guidelines.xhtml

are those that fall into the 10% most deprived practice populations in England. These practices have between 95.7% and 57.7% of registered patients living in the most deprived 15% of indices of multiple deprivation (IMD) data zones. Due to the high rates of long term conditions, unhealthy diets and physical inactivity, together with other competing priorities [13], people in areas of deprivation are likely to face greater challenges in managing CVDs. Ongoing effects from the pandemic are exacerbating these challenges and include difficulties attending review appointments in person, digital poverty impeding remote review, low levels of health literacy resulting in misunderstandings about the need to continue long term treatments, and closure of other support services [14], potentially widening health inequalities.

The NHS has set up the national CVD Prevention programme [15] which aims to develop targeted interventions to minimise risk factors by maximising diagnosis and treatment, accompanied by the GP contract to commission a new national CVD prevention audit for primary care [16]. However, no evidence is available on what and how the intervention has been implemented and for what health outcomes for deprived populations. There is therefore an urgent need to seek a theoretical underpinning to tailor the national programme in this context, which could support the CVD element of the NHS post-COVID-19 recovery plan with the region.

This study will examine the literature and practice-level data and undertake engagement with staff who provide primary care for deprived populations to define the components and mechanisms through which lipid management can be optimised to meet the identified needs. The study aims to 1) synthesise the evidence on interventions for deprived populations with CVDs or those with high risks and understand the outcomes associated with these interventions, 2) assess CVD risk management for deprived populations in the NENC in comparison with non-deprived populations in Northern England and with England overall in order to identify clinical gaps and needs, 3) investigate the implementation and delivery of current interventions for patients with CVDs and those with high risks, and 4) tailor and optimise the national prevention programme to suit the context and needs of deprived communities.

METHODS AND ANALYSIS

Study design

A mixed methods approach will be employed to inform the development of the intervention comprising a rapid review, a population based observational study and qualitative interviews. Four work packages (WPs) are proposed (Figure 1).

[insert Figure 1]

For peer review only - http://bmjopen.bmj.com/site/about/guidelines.xhtml

A project advisory group consisting of 6-8 members will be established to involve key opinion leaders across core fields, who will advise at each project stage, review intervention components for the consensus process and help disseminate the study outputs. Members will recruit from the Deep End network, ICS Prevention Board, Academic Health Science Network (AHSN) NENC lipid steering group, regional professional leads for lipid management, public members and academics with methodology expertise. This group will meet quarterly with the research team to oversee the execution of the study and provide advice and assistance.

Patient and public involvement

Patients' experiences are central to the research question and outcomes, although the focus of this project is on clinicians' experiences. The Deep End Steering group, consisting of local GPs, representatives from the North East Commissioning Support Unit (NECS), Newcastle University Medical School, Health Education England North East and the Postgraduate School of Primary Care, Directors of Public Health, NHS England, the Northern Cancer Alliance and local voluntary, community and social enterprise organisations, was consulted in order to shape the research focus and question, methods of data collection and dissemination. While the focus of the research is initially on clinicians' experience, the development of a patient and public involvement strategy was recognised in the consultation as an urgent requirement, and this is currently being developed. This results of this study will be widely shared via the Public Involvement and Community Engagement network for the National Institute for Health Research (NIHR) Applied Research Collaboration NENC.

WP1: Rapid review and logic model (months 1-3)

WP 1 will be a rapid review and synthesis of current evidence, aiming to identify evidence-based interventions for lipid management in deprived areas and targeted outcomes. The review will be conducted following Cochrane guidance on rapid reviews [17]. A logic model will be developed, informed by existing literature to describe how lipid management works in theory to benefit services and patients.

Type of studies

Empirical studies (i.e. original data collection) describing the setting, problem addressed, resource requirements, aim, intervention components, provider, method of delivery and objective and subjective outcomes will be included if conducted in an Organisation for Economic Co-operation and Development (OECD) country [18] (to ensure a degree of commonality in health system and socioeconomic and demographic context), published in peer-reviewed scientific journals, within the last 10 years (to mirror the NHS long term plan) and in the English language.

For peer review only - http://bmjopen.bmj.com/site/about/guidelines.xhtml

For peer review only

Type of participants

Studies that focus on people with disadvantaged socioeconomic status (education, income, occupation, social class, deprivation, poverty or an area-based proxy for deprivation derived from place of residence) will be included. Adults with CVD including angina, previous myocardial infarction (MI), revascularisation, stroke or TIA or symptomatic peripheral arterial disease, and those who do not have established CVD but are identified as having a high risk of developing CVDs [1] considering age, ethnicity, socioeconomic status, BMI, history of taking antihypertensive or lipid modification therapy, QRISK$\geq$10%, diabetes, nephropathy, familial hypercholesterolaemia or other inherited disorder of lipid metabolism, and other underlying medical conditions or treatment including people treated for HIV, with serious mental health problems, taking medicines that can cause dyslipidaemia or with autoimmune disorders.

Type of interventions

Multifaceted interventions delivered to deprived populations that aim to optimise care by maximising diagnosis and/or treatment to minimise individual risk factors will be considered.

Type of outcome measures

Studies with individual, area-based or both types of measures of socioeconomic deprivation will be included. This may be measured according to several characteristics including income, employment, education, disability, crime, housing and services and living environment deprivation. Because there is no universal recommendation for core outcome sets in studies on CVD prevention [19-21], studies will be eligible for inclusion regardless of outcomes measured or reported for health outcomes, this may include vascular-related outcomes, cognitive and functional outcomes, lifestyle, medical risk factors, cardioprotective medications and patient-reported outcome measures. Any measures of professionals', patients' and/or families' knowledge, attitudes or satisfaction will also be included.

Study identification

Cochrane CENTRAL, MEDLINE, PsycInfo, CINAHL will be searched for eligible studies. Detailed search strategies will be developed for each database. A preliminary search strategy developed for MEDLINE is designed by YF and validated by an information specialist (supplementary material 1). This search strategy was piloted in MEDLINE on 16th October 2021.

Study selection

Identified citations will be exported to Endnote X9 [22] for deduplication and screening. A random selection (10%) of study titles and abstracts will be screened independently by another researcher. Full text will be retrieved where citations appeared to meet the eligibility criteria or where a decision

For peer review only - http://bmjopen.bmj.com/site/about/guidelines.xhtml

to exclude will not be made on the information provided. Any discrepancies will be resolved by discussion with a third researcher.

Data extraction

Data will be extracted on author's first name, publication date, location (country in which the study was undertaken), study design, sample size, intervention details, control/comparison groups (if any), outcome measures and results, using a data extraction sheet that will be piloted on two retrieved study reports. Accuracy and consistency will be monitored through random double-extraction of 10% included studies by an independent researcher. Any discrepancies will be resolved by discussion with a third researcher. Where a study appears to have multiple citations, original authors will be contacted for clarification. All information from multiple citations will be used if no replies received.

Quality assessment

Quality appraisal of included studies will be performed using standardised tools adapted for purpose. Appropriate Critical Appraisal Skills Programme (CASP) tool will be used according to the study design, a random sample (10%) will be independently assessed by another researcher. Any discrepancies will be resolved by discussion with a third researcher.

Data synthesis

A narrative synthesis will be undertaken following Popay et al's [23] approach to conducting synthesis systematically and transparently. It will focus on the intervention components, effects of the interventions and mechanisms leading to the outcomes. Studies, interventions and outcomes will be examined and grouped according to the aim and components of the interventions. The variation on different characteristics of health systems will be taken into account when interpreting the intervention across OECD countries. A logic model will be produced to present context, intervention components and outcomes. Possible unintended adverse outcomes will also be provided.

WP2: Assessment and comparison of CVD risk management for deprived with non-deprived populations to England overall (months 2-4)

WP2 will be a population based observational study comparing retrospective data from practices in deprived communities in the NENC, practices in regional non-deprived communities and national practice-level data obtained from publicly accessible datasets and anonymised data requested from the NECS that securely house primary and secondary care datasets.

Data sources

The primary data source for this study will be the GP Practice Profiles [24] via Fingertips, a publicly accessible web tool containing national general practice profiles generated for all Quality and

For peer review only - http://bmjopen.bmj.com/site/about/guidelines.xhtml

Outcomes Framework (QOF)[25] 2019/20 with a list size of at least 750 patients. Available practice-level data include local demography, QOF domains and patient satisfaction. Other data sources used include the QOF, OpenPerscribing[26] and data requested from the NECS via Secondary Uses Services (SUS)[27] data (Table 1).

Table 1 Data sources and variables

Data source	Description	Level of data available	Variables and variable description
GP Practice Profiles	Date reported by GPs to the NHS that refers to all patients in a practice	• Individual practice	• Practice size<br>• Mean practice age<br>• Deprivation score<br>• Age groups<br>• Percentage of patients positive experiences as "good"<br>• Percentage of practice access rated by patients as "good"<br>• Percentage with a long term condition<br>• Education status<br>• Working status<br>• Life expectancy by sex
QOF	An indication of the overall achievement of a practice through a points system, concerning clinical, public health, public health – additional services, and quality improvement.<br>It also has cardiovascular group data.	• Individual practice	• QOF score<br>• Total on the atrial fibrillation (AF) register, prevalence<br>• Total on the CVD-primary prevention (CVD-PP) register, prevalence<br>• Total on the CHD register, prevalence<br>• Total on the heart failure (HF) register, prevalence<br>• Total on the left ventricular systolic dysfunction (LVSD) register, prevalence<br>• Total on the hypertension (HYP) register, prevalence<br>• Total on the peripheral arterial disease (PAD) register, prevalence<br>• Total on the stroke and transient ischaemic attack (STIA) register, prevalence<br>•
OpenPrescribing	Imports national prescribing data published by NHS Business Services Authority	• Individual practice<br>• CCG level	• Total statin<br>• Total low and medium intensity statin

For peer review only - http://bmjopen.bmj.com/site/about/guidelines.xhtml

Study population

The study population are patients aged 16 and above who have registered with the 34 Deep End practices in the NENC and have been diagnosed with any form of CVDs recorded on the QOF from 2019 to 2020. The study comparators are the patients registered in non-Deep End practices in the region and all registered patients in England where data is available. Data is aggregated to the GP practice level in which variables will be summarised if they are at the patient level.

Data analysis

Descriptive analysis will be used to provide a quick and low cost approach to assess CVD risk management and give descriptive statistics or investigate relationships between factors. GP practice code will be used to link data across all datasets.

Descriptive statistics, using means, standard deviations, and range, will be used to compare the practice profile of the 34 Deep End practices with non-Deep End in the region and England average level. The prevalence of risk factors and statin prescribing will be analysed with an appropriate statistical test (i.e., two-sample t-test, single sample t-test, and paired t-test), which will yield p values that indicate the statistical significance of any differences between Deep End, non-Deep End and England level, and over time. Confidence intervals for differences in means, medians or percentages will be calculated. All significance tests will be performed at the 5% level. Stata version 16 will be used to facilitate data analysis.

WP3: Interviews with health professionals (months 3-8)

WP3 will be qualitative interviews with staff involved in the organisation and delivery of routine lipid management in practices that are part of the Deep End Network. The aim of the interviews will be to understand the implementation and delivery of current lipid management and identify their needs and challenges.

Participants and recruitment

All health professionals involved in the management of CVDs in the practice are eligible to take part including GPs, pharmacists, assistant practitioners, practice nurses and social prescribers.

Study recruitment will be supported by the Deep End practice Network, who will send an email containing brief study information to healthcare professionals working in participating practices. Health professionals can express their interests by responding to the email straight to the research team. A reminder will be sent to those who have not responded in two weeks. Maximum variation sampling will be used to ensure a broad representation of health professionals on dimensions

For peer review only - http://bmjopen.bmj.com/site/about/guidelines.xhtml

including job titles/roles, grade, speciality, length of working, and demographics. Reasons given by practices for declining to participate will be recorded to inform feasibility assessment to further studies.

Data collection

With participants' informed written consent, semi-structured interviews will be conducted via telephone or online (i.e. Zoom or MS Teams) for up to 60 mins. A topic guide was drafted to address the research questions and piloted with two primary care health professionals to ensure the questions prepared are relevant for the context and acceptable. Questions considered important but not originally included were also sought from the pilot interviews, and the topic guide was amended accordingly (supplementary material 2). As interviews continue, the topic guide will also allow a deeper exploration of emerging themes and participants' feedback, while maintaining a consistent core of questions. Data collection will end when data saturation is reached indicating no new information is discovered.

Data analysis

Interviews will be digitally recorded (with consent), transcribed and data managed using NVivo 12, a qualitative software programme to assist with the organisation and coding of data. Data will be analysed using Framework Analysis, which provides a systematic approach to sifting, charting, and sorting material using the key themes and issues. Initial line-by-line coding will be undertaken. The connections and relationships of these codes will be explored, contributing to the development of themes. An analytical framework will have been developed as the coding process progresses and themes emerges. Codes and themes from each transcript will be compared and integrated using the constant comparison process, enabling continuous updates on the interview topic guide and the thorough interpretation of the study data. To ensure trustworthiness and rigour of the analysis, the coding framework will be developed and assured by double coding of a random sample of transcripts (10%) as a validity check and exploring alternative interpretations of the data.

WP4: Intervention development (months 7-9)

WP 4 will develop the prototype of the intervention in collaboration with the Academic Health Science Network NENC which delivers the CVD Prevention programme, part of which includes a national programme mandated by NHS England and NHS Improvement.

Guided by the MRC Framework for developing and evaluating complex interventions [28], the development of intervention will be informed by integrating the outcomes of the literature evidence, current CVD management profile, and stakeholder engagement undertaken in WPs1-3, in

For peer review only - http://bmjopen.bmj.com/site/about/guidelines.xhtml

an iterative and progressive approach. The national programme and its key components will be examined against the gaps, needs, and challenges identified to consider the wider context. The prototype intervention will be designed taking account of health professionals' existing commitments in these practices and challenging working environments. Training and skills development materials for health professionals will also be developed to facilitate them in delivering the tailored intervention. The logic model produced in WP1 will be refined to map key intervention processes and outcomes.

Design

The prototype intervention will be reviewed and assessed by the project advisory group, guided by a nominal group technique, a consensus method that allows for the generation of views and thoughts from group participants whilst maintaining anonymity throughout [29].

Data collection

The group will be provided with details of the interventions and refined logic model, to seek further comments and explore if the intervention is feasible, acceptable, and implementable in the context. The APEASE criteria [30] will be used to determine the acceptability, practicability, effectiveness, affordability, side-effects, and equity aspects of the intervention. The nominal group technique will involve two main sections:

1. The group will be asked to provide their comments on the intervention, training materials, and logic model. All comments will be collated and grouped into main themes for each member to rate their top 10 priorities of the comments. Group ratings will be summated, and the group's collective top 10 priorities will be presented to the group and discussed.
2. Each will re-rate the group's top 10 priorities and provide a weighting for their top 10 comments in the scale ranging from 1=least important to 100=most important. These weightings will be summated after the meeting, which will be used to refine the intervention and the logic model. The refined version will be sent out to each member for further comments. It is expected that this process will be repeated twice until a census is reached.

Data analysis

The initial listing of comments, clarification, and discussion of comments in section 1 will be analysed thematically, with further discussion with the research team. The scale data generated in section 2 will be averaged so that the comments can be re-ordered according to weighted ranked priority. The individual/group rankings produced in sections 1 and 2 will be compared to estimate the level of agreement between the sections and to observe the process of reaching consensus. Firstly, these comparisons will be made by calculating the percentage agreement between the sections, in terms of

For peer review only - http://bmjopen.bmj.com/site/about/guidelines.xhtml

the comments that appear in the top ten priorities each time. Secondly, the movement in ranking between sections 1 and 2 will be estimated using Cohen's kappa statistic of chance-corrected agreement [31]. A kappa value of >0.40 is considered to represent a moderate level of agreement [32].

ETHICS AND DISSEMINATION

Ethics approval has been sought from the Faculty of Medical Sciences Research Ethics Committee at Newcastle University (reference no.: 2209/14251) UK, a letter of support has also been issued by the NENC Deep End Network.

Dissemination will be led by the research team and supported by the project advisory group. Reports will be produced and shared with the NENC ICS Prevention Board, Inequalities Board, Deep End network, National Institute for Health Research (NIHR) Applied Research Collaboration (ARC) NENC, and AHSN NENC. The findings will be disseminated to the participating sites, participants, commissioners, and in peer-reviewed journals and academic conferences.

For peer review only

For peer review only - http://bmjopen.bmj.com/site/about/guidelines.xhtml

FIGURES

Figure 1 Study design and related work packages

ACKNOWLEDGEMENT

We thank NENC Deep End Steering Group and NECSU for their support in this study.

AUTHORS' CONTRIBUTIONS

YF, JN and PW conceived the study design. YF drafted and revised the manuscript and obtained the ethics approval. EYHT, SS, JN and PW independently reviewed and contributed to revising and approving the final version.

FUNDING

This project/research/work is supported by the National Institute of Health Research (NIHR) [Applied Research Collaboration North East and North Cumbria (NIHR200173)]. The views expressed are those of the author(s) and not necessarily those of the NIHR or the Department of Health and Social Care.

EYHT (National Institute for Health Research (NIHR) Clinical Lecturer) is funded by the NIHR. The views expressed in this publication are those of the author(s) and not necessarily those of the NIHR, NHS or the UK Department of Health and Social Care.

COMPETING INTERESTS

None declared.

For peer review only

REFERENCES

1. NICE. Cardiovascular disease: risk assessment and reduction, including lipid modification National Institute for Health and Care Excellence; 2014 [accessed 19th July 2021.
2. Eurostat. Cardiovascular diseases statistics: Eurostat; 2020 [Available from: https://ec.europa.eu/eurostat/statistics-explained/index.php?title=Cardiovascular_diseases_statistics#Deaths_from_cardiovascular_diseases accessed 20th July 2021.
3. Nelson RH. Hyperlipidemia as a risk factor for cardiovascular disease. *Prim Care* 2013;40(1):195-211. doi: 10.1016/j.pop.2012.11.003 [published Online First: 2012/12/04]
4. Public Health England. Health matters: preventing cardiovascular disease: Public Health England; 2019 [Available from: https://www.gov.uk/government/publications/health-matters-preventing-cardiovascular-disease/health-matters-preventing-cardiovascular-disease accessed 20th July 2021.
5. Mach F, Baigent C, Catapano AL, et al. 2019 ESC/EAS Guidelines for the management of dyslipidaemias: lipid modification to reduce cardiovascular risk: The Task Force for the management of dyslipidaemias of the European Society of Cardiology (ESC) and European Atherosclerosis Society (EAS). *European Heart Journal* 2019;41(1):111-88. doi: 10.1093/eurheartj/ehz455
6. Wu J, Zhu S, Yao GL, et al. Patient Factors Influencing the Prescribing of Lipid Lowering Drugs for Primary Prevention of Cardiovascular Disease in UK General Practice: A National Retrospective Cohort Study. *PLOS ONE* 2013;8(7):e67611. doi: 10.1371/journal.pone.0067611
7. Ray KK, Molemans B, Schoonen WM, et al. EU-Wide Cross-Sectional Observational Study of Lipid-Modifying Therapy Use in Secondary and Primary Care: the DA VINCI study. *European Journal of Preventive Cardiology* 2020 doi: 10.1093/eurjpc/zwaa047
8. NHS. Optimising treatment of high-risk conditions: NHS; 2021 [Available from: https://www.england.nhs.uk/ltphimenu/cvd/optimising-treatment-of-high-risk-conditions/ accessed 28th September 2021.
9. Lang S-J, Abel GA, Mant J, et al. Impact of socioeconomic deprivation on screening for cardiovascular disease risk in a primary prevention population: a cross-sectional study. *BMJ Open* 2016;6(3):e009984. doi: 10.1136/bmjopen-2015-009984
10. NHS. The NHS Long Term Plan London: National Health Service; 2019 [Available from: https://www.longtermplan.nhs.uk/wp-content/uploads/2019/08/nhs-long-term-plan-version-1.2.pdf accessed 20th July 2021.
11. Corris V, Dormer E, Brown A, et al. Health inequalities are worsening in the North East of England. *British Medical Bulletin* 2020;134(1):63-72. doi: 10.1093/bmb/ldaa008
12. Watt G, Brown G, Budd J, et al. General Practitioners at the Deep End: The experience and views of general practitioners working in the most severely deprived areas of Scotland. *Occasional paper (Royal College of General Practitioners)* 2012(89):i.
13. Holmes J. Tackling obesity: the role of the NHS in a whole-system approach: The King's Fund; 2021 [Available from: https://www.kingsfund.org.uk/sites/default/files/2021-07/Tackling%20obesity.pdf accessed 20th July 2021.
14. Norman C, Wildman JM, Sowden S. COVID-19 at the Deep End: A Qualitative Interview Study of Primary Care Staff Working in the Most Deprived Areas of England during the COVID-19 Pandemic. *International Journal of Environmental Research and Public Health* 2021;18(16):8689.
15. NHS England. Cardiovascular disease London: National Health Service,; 2019 [Available from: https://www.england.nhs.uk/ourwork/clinical-policy/cvd/ accessed 20th July 2021.
16. NHS England. CVDPREVENT London: National Health Service; 2019 [Available from: https://www.england.nhs.uk/ourwork/clinical-policy/cvd/cvdprevent/ accessed 20th July 2021.

For peer review only - http://bmjopen.bmj.com/site/about/guidelines.xhtml

17. Garritty C, Gartlehner G, Kamel C, et al. Cochrane Rapid Reviews Interim Guidance from the Cochrane Rapid Reviews Methods Group. March 2020, 2020.
18. Reibling N, Ariaans M, Wendt C. Worlds of Healthcare: A Healthcare System Typology of OECD Countries. *Health Policy* 2019;123(7):611-20. doi: https://doi.org/10.1016/j.healthpol.2019.05.001
19. McNamara RL, Spatz ES, Kelley TA, et al. Standardized Outcome Measurement for Patients With Coronary Artery Disease: Consensus From the International Consortium for Health Outcomes Measurement (ICHOM). *Journal of the American Heart Association* 2015;4(5):e001767. doi: doi:10.1161/JAHA.115.001767
20. Neilson LM, Swart ECS, Good CB, et al. Identifying Outcome Measures for Coronary Artery Disease Value-Based Contracting Using the Delphi Method. *Cardiology and Therapy* 2019;8(1):135-43. doi: 10.1007/s40119-019-0132-7
21. Canavan M, Smyth A, Robinson SM, et al. Attitudes to outcomes measured in clinical trials of cardiovascular prevention. *QJM: An International Journal of Medicine* 2015;109(6):391-97. doi: 10.1093/qjmed/hcv132
22. Hupe M. EndNote X9. *Journal of Electronic Resources in Medical Libraries* 2019;16(3-4):117-19. doi: 10.1080/15424065.2019.1691963
23. Popay J, Roberts H, Sowden A, et al. Guidance on the conduct of narrative synthesis in systematic reviews. *A product from the ESRC methods programme Version* 2006;1:b92.
24. Public Health England. Public Health Profiles.: Public Health England; 2021 [Available from: https://fingertips.phe.org.uk accessed 29th Oct 2021.
25. NHS Digital. Quality Outcomes Framework (QOF): NHS Digital; 2021 [accessed 29th October 2021.
26. OpenPrescribing.net. EBM DataLab, University of Oxford, 2017.
27. NHS Digital. Secondary Uses Service (SUS): NHS Digital; 2021 [Available from: https://digital.nhs.uk/services/secondary-uses-service-sus accessed 29th Oct 2021.
28. Craig P, Dieppe P, Macintyre S, et al. Developing and evaluating complex interventions: the new Medical Research Council guidance. *Bmj* 2008;337
29. Harvey N, Holmes CA. Nominal group technique: an effective method for obtaining group consensus. *International journal of nursing practice* 2012;18(2):188-94.
30. West R, Michie S, Atkins L, et al. Achieving behaviour change: A guide for local government and partners London: Public Health England; 2019 [Available from: https://assets.publishing.service.gov.uk/government/uploads/system/uploads/attachment_data/file/875385/PHEBI_Achieving_Behaviour_Change_Local_Government.pdf accessed 14th September 2021.
31. Cohen J. A coefficient of agreement for nominal scales. *Educational and Psychological Measurement* 1960
32. Landis JR, Koch GG. The measurement of observer agreement for categorical data. *biometrics* 1977:159-74.

For peer review only - http://bmjopen.bmj.com/site/about/guidelines.xhtml

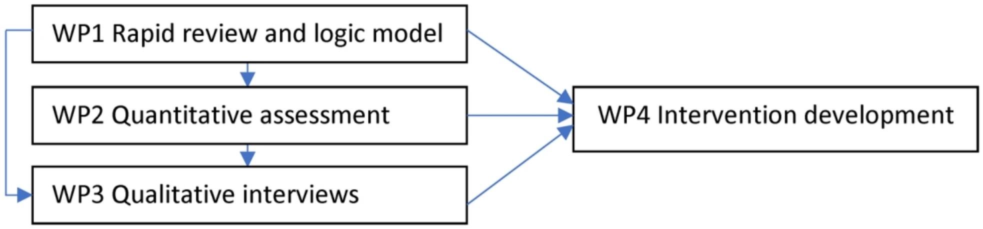

Figure 1 Study design and related work packages

139x34mm (300 x 300 DPI)

Supplementary Material 1 Search strategy for MEDLINE (Ovid) (1996 to October Week 4 2021)

1. exp Cardiovascular Diseases/
2. cardio*.tw.
3. cardia*.tw.
4. heart*.tw.
5. coronary*.tw.
6. angina*.tw.
7. ventric*.tw.
8. myocard*.tw.
9. pericard*.tw.
10. isch?em*.tw.
11. emboli*.tw.
12. arrhythmi*.tw.
13. thrombo*.tw.
14. atrial fibrillat*.tw.
15. tachycardi*.tw.
16. endocardi*.tw.
17. (sick adj sinus).tw.
18. exp Stroke/

For peer review only

For peer review only - http://bmjopen.bmj.com/site/about/guidelines.xhtml

19. (stroke or strokes).tw.
20. cerebrovasc*.tw.
21. cerebral vascular.tw.
22. apoplexy.tw.
23. ((brain* or cerebral or lacunar) adj2 infarct*).tw.
24. exp Hypertension/
25. hypertensi*.tw.
26. peripheral arter* disease*.tw.
27. ((high or increased or elevated) adj2 blood pressure).tw.
28. exp Hyperlipidemias/
29. hyperlipid*.tw.
30. hyperlip?emia*.tw.
31. hypercholesterol*.tw.
32. hypercholester?emia*.tw.
33. hyperlipoprotein?emia*.tw.
34. hypertriglycerid?emia*.tw.
35. exp Arteriosclerosis/
36. exp Cholesterol/
37. cholesterol.tw.
38. Blood Pressure/

For peer review only

For peer review only - http://bmjopen.bmj.com/site/about/guidelines.xhtml

39. 1 or 2 or 3 or 4 or 5 or 6 or 7 or 8 or 9 or 10 or 11 or 12 or 13 or 14 or 15 or 16 or 17 or 18 or 19 or 20 or 21 or 22 or 23 or 24 or 25 or 26 or 27 or 28 or 29 or 30 or 31 or 32 or 33 or 34 or 35 or 36 or 37 or 38

40. exp Socioeconomic Factors/

41. exp social class/

42. socioecon*.tw.

43. demographic*.tw.

44. disadvantage*.tw.

45. disparit*.tw.

46. deprivation.tw.

47. exp Health Services Accessibility/

48. deprive*.tw.

49. pover*.tw.

50. inequalit*.tw.

51. education*.tw.

52. unemploy*.tw.

53. employed.tw.

54. employment.tw.

55. income.tw.

56. occupation*.tw.

57. SES.tw.

For peer review only - http://bmjopen.bmj.com/site/about/guidelines.xhtml

58. class.tw.

59. economic.tw.

60. (social adj1 (class or factor or factors)).tw.

61. 40 or 41 or 42 or 43 or 44 or 45 or 46 or 47 or 48 or 49 or 50 or 51 or 52 or 53 or 54 or 55 or 56 or 57 or 58 or 59 or 60

62. exp General Practitioners/

63. exp General Practice/

64. general pract*.tw.

65. exp Family Practice/

66. family pract*.tw.

67. family medicine/

68. exp Family Practice/

69. exp Physicians, Family/

70. family phys*.tw.

71. exp Nurse Practitioners/

72. exp Nurse Clinicians/

73. nurse pract*.tw.

74. nurse clinic*.tw.

75. exp Primary Health Care/

76. primary care.tw.

77. exp Community Health Services/

For peer review only - http://bmjopen.bmj.com/site/about/guidelines.xhtml

For peer review only

78. community care.tw.

79. 62 or 63 or 64 or 65 or 66 or 67 or 68 or 69 or 70 or 71 or 72 or 73 or 74 or 75 or 76 or 77 or 78

80. 39 and 61 and 79

81. (Algeria$ or Egypt$ or Liby$ or Morocc$ or Tunisia$ or Western Sahara$ or Angola$ or Benin or Botswana$ or Burkina Faso or Burundi or Cameroon or Cape Verde or Central African Republic or Chad or Comoros or Congo or Djibouti or Eritrea or Ethiopia$ or Gabon or Gambia$ or Ghana or Guinea or Keny$ or Lesotho or Liberia or Madagasca$ or Malawi or Mali or Mauritania or Mauritius or Mayotte or Mozambiq$ or Namibia$ or Niger or Nigeria$ or Reunion or Rwand$ or Saint Helena or Senegal or Seychelles or Sierra Leone or Somalia or South Africa$ or Sudan or Swaziland or Tanzania or Togo or Ugand$ or Zambia$ or Zimbabw$ or China or Chinese or Hong Kong or Macao or Mongolia$ or Taiwan$ or Belarus or Moldov$ or Russia$ or Ukraine or Afghanistan or Armenia$ or Azerbaijan or Bahrain or Cyprus or Cypriot or Georgia$ or Iran$ or Iraq$ or Israel$ or Jordan$ or Kazakhstan or Kuwait or Kyrgyzstan or Leban$ or Oman or Pakistan$ or Palestin$ or Qatar or Saudi Arabia or Syria$ or Tajikistan or Turkmenistan or United Arab Emirates or Uzbekistan or Yemen or Bangladesh$ or Bhutan or British Indian Ocean Territory or Brunei Darussalam or Cambodia$ or India$ or Indonesia$ or Lao or People's Democratic Republic or Malaysia$ or Maldives or Myanmar or Nepal or Philippin$ or Singapore or Sri Lanka or Thai$ or Timor Leste or Vietnam or Albania$ or Andorra or Bosnia$ or Herzegovina$ or Bulgaria$ or Croatia$ or Estonia or Faroe Islands or Greenland or Liechtenstein or Lithuani$ or Macedonia or Malta or maltese or Romania or Serbia$ or Montenegro or Slovenia or Svalbard or Argentina$ or Belize or Bolivia$ or Brazil$ or chile or Chilean or Colombia$ or Costa Rica$ or Cuba or Ecuador or El Salvador or French Guiana or Guatemala$ or Guyana or Haiti or Honduras or Jamaica$ or Nicaragua$ or Panama or Paraguay or Peru or Puerto Rico or Suriname or Uruguay or Venezuela or developing countr$ or south America$).ti,sh. [mp=title, abstract, original title, name of substance word, subject heading word, floating sub-heading word, keyword heading word, organism supplementary concept word, protocol supplementary concept word, rare disease supplementary concept word, unique identifier, synonyms]

82. 80 not 81

83. limit 82 to (abstracts and english language and humans and yr="2011 -Current")

For peer review only - http://bmjopen.bmj.com/site/about/guidelines.xhtml

Interview topic guide

This interview is to learn from your experience on what and how lipid management is currently delivered to patients in your practice. The aim is to understand the implementation process and resources available for the existing interventions targeting patients with cardiovascular disease (CVD) and/or risks, as well as your needs and any challenges to providing lipid management optimisation in deprived communities.

The interview will be recorded with your permission. It will last for about 45-60 minutes and any information you give will be kept confidential and anonymous.

Our conversation will cover:

- Your job title, an overview of patients in your practice, what your role involves for patients with CVD risks;
- Your understanding of lipid management and what it involves;
- How patients with CVD (or CVD risks) are managed and their clinical pathways in your practice (screening/assessment/monitoring/treatment/outcomes/referrals):
    - How you identify patients with CVD risks (e.g. NHS Health Check; frequency), and is there anything else that would indicate the need for care for you;
    - What assessment tool(s) you use to understand their needs;
    - How you monitor their disease progress;
    - Existing treatment and care provided for patients and rationale for that;
    - Clinically meaningful outcomes;
    - How and when you decide on referrals and for what services.
- Your views on the implementation process of current interventions targeting patients with CVD in your practice, and what resource is needed for their delivery;
- Your experience/challenges of managing patients' needs, and factors that influence the delivery and quality of lipid management to patients;
- The impact of COVID on lipid management in your practice and solutions implemented;
- To what extent you have accessed training (general/lipid) to support your clinical role; other information, skills and training that you may need to help you optimise lipid management;
- Any other questions or comments and suggestions that you want to mention on how you provide services for patients with CVD and what additional support you might need;
- Any other questions you have about this study.

For peer review only - http://bmjopen.bmj.com/site/about/guidelines.xhtml

PRISMA-P (Preferred Reporting Items for Systematic review and Meta-Analysis Protocols) 2015 checklist: recommended items to address in a systematic review protocol*

Section and topic	Item No	Checklist item	Page no.
**ADMINISTRATIVE INFORMATION**			
Title:			
Identification	1a	Identify the report as a protocol of a systematic review	
Update	1b	If the protocol is for an update of a previous systematic review, identify as such	
Registration	2	If registered, provide the name of the registry (such as PROSPERO) and registration number	
Authors:			
Contact	3a	Provide name, institutional affiliation, e-mail address of all protocol authors; provide physical mailing address of corresponding author	
Contributions	3b	Describe contributions of protocol authors and identify the guarantor of the review	
Amendments	4	If the protocol represents an amendment of a previously completed or published protocol, identify as such and list changes; otherwise, state plan for documenting important protocol amendments	
Support:			
Sources	5a	Indicate sources of financial or other support for the review	
Sponsor	5b	Provide name for the review funder and/or sponsor	
Role of sponsor or funder	5c	Describe roles of funder(s), sponsor(s), and/or institution(s), if any, in developing the protocol	
**INTRODUCTION**			
Rationale	6	Describe the rationale for the review in the context of what is already known	5-6
Objectives	7	Provide an explicit statement of the question(s) the review will address with reference to participants, interventions, comparators, and outcomes (PICO)	6
**METHODS**			
Eligibility criteria	8	Specify the study characteristics (such as PICO, study design, setting, time frame) and report characteristics (such as years considered, language, publication status) to be used as criteria for eligibility for the review	7-8
Information sources	9	Describe all intended information sources (such as electronic databases, contact with study authors, trial registers or other grey literature sources) with planned dates of coverage	8
Search strategy	10	Present draft of search strategy to be used for at least one electronic database, including planned limits, such that it could be	Supplementary

For peer review only

		repeated	
Study records:			
Data management	11a	Describe the mechanism(s) that will be used to manage records and data throughout the review	8
Selection process	11b	State the process that will be used for selecting studies (such as two independent reviewers) through each phase of the review (that is, screening, eligibility and inclusion in meta-analysis)	8
Data collection process	11c	Describe planned method of extracting data from reports (such as piloting forms, done independently, in duplicate), any processes for obtaining and confirming data from investigators	8
Data items	12	List and define all variables for which data will be sought (such as PICO items, funding sources), any pre-planned data assumptions and simplifications	9
Outcomes and prioritization	13	List and define all outcomes for which data will be sought, including prioritization of main and additional outcomes, with rationale	9
Risk of bias in individual studies	14	Describe anticipated methods for assessing risk of bias of individual studies, including whether this will be done at the outcome or study level, or both; state how this information will be used in data synthesis	8-9
Data synthesis	15a	Describe criteria under which study data will be quantitatively synthesised	9
	15b	If data are appropriate for quantitative synthesis, describe planned summary measures, methods of handling data and methods of combining data from studies, including any planned exploration of consistency (such as I^2, Kendall's τ)	
	15c	Describe any proposed additional analyses (such as sensitivity or subgroup analyses, meta-regression)	
	15d	If quantitative synthesis is not appropriate, describe the type of summary planned	
Meta-bias(es)	16	Specify any planned assessment of meta-bias(es) (such as publication bias across studies, selective reporting within studies)	n/a
Confidence in cumulative evidence	17	Describe how the strength of the body of evidence will be assessed (such as GRADE)	n/a

*** It is strongly recommended that this checklist be read in conjunction with the PRISMA-P Explanation and Elaboration (cite when available) for important clarification on the items. Amendments to a review protocol should be tracked and dated. The copyright for PRISMA-P (including checklist) is held by the PRISMA-P Group and is distributed under a Creative Commons Attribution Licence 4.0.**

From: Shamseer L, Moher D, Clarke M, Ghersi D, Liberati A, Petticrew M, Shekelle P, Stewart L, PRISMA-P Group. Preferred reporting items for systematic review and meta-analysis protocols (PRISMA-P) 2015: elaboration and explanation. BMJ. 2015 Jan 2;349(jan02 1):g7647.

For peer review only

Tailoring lipid management interventions to reduce inequalities in cardiovascular disease risk management in primary care for deprived communities in Northern England: a mixed methods intervention development protocol

Journal:	*BMJ Open*
Manuscript ID	bmjopen-2021-058951.R2
Article Type:	Protocol
Date Submitted by the Author:	27-May-2022
Complete List of Authors:	Fu, Yu; Newcastle University Faculty of Medical Sciences, Population Health Sciences Institute<br>Tang, Eugene; Newcastle University Faculty of Medical Sciences<br>Sowden, Sarah; Newcastle University, Institute Health and Society;<br>Newton, Julia L.; Newcastle University<br>Whitty, Paula; Cumbria Northumberland Tyne and Wear NHS Foundation Trust
**Primary Subject Heading**:	Cardiovascular medicine
Secondary Subject Heading:	General practice / Family practice, Public health, Cardiovascular medicine
Keywords:	Cardiology < INTERNAL MEDICINE, PRIMARY CARE, PUBLIC HEALTH

SCHOLARONE™
Manuscripts

Tailoring lipid management interventions to reduce inequalities in cardiovascular disease risk management in primary care for deprived communities in Northern England: a mixed methods intervention development protocol

Y Fu*[1,2], EYH Tang[2], S Sowden[2], J Newton[2,3], P Whitty[1]

1. National Institute for Health Research ARC North East and North Cumbria, Population Health Sciences Institute, Newcastle University, Newcastle, UK

2. Population Health Sciences Institute, Newcastle University, Newcastle, UK

3. Academic Health Science Network for the North East and North Cumbria, Newcastle, UK

*Correspondence

Dr Yu Fu

Baddiley-Clark Building

Richardson Road

Population Health Sciences Institute

Newcastle University

Newcastle

NE2 4AX

yu.fu@newcastle.ac.uk

01912 085307

Keywords: health inequalities; deprived populations; lipid management; mixed methods; protocol

Word count: 3500

For peer review only - http://bmjopen.bmj.com/site/about/guidelines.xhtml

ABSTRACT

Introduction

Hyperlipidaemia contributes a significant proportion of modifiable cardiovascular disease (CVD) risk, which is a condition that disproportionally affects disadvantaged socioeconomic communities, with death rates in the most deprived areas being four times higher than those in the least deprived. With the national CVD Prevention programme being delivered to minimise risk factors, no evidence is available on what has been implemented in primary care for deprived populations. This study describes the protocol for the development of a tailored intervention aiming to optimise lipid management in primary care settings to help reduce inequalities in CVD risks and improve outcomes in deprived communities.

Methods and analysis

A mixed methods approach will be employed consisting of four workpackages: 1) rapid review and logic model; 2) assessment and comparison of CVD risk management for deprived with non-deprived populations in Northern England to England overall; 3) interviews with health professionals; 4) intervention development. A systematic search and narrative synthesis will be undertaken to identify evidence-based interventions and targeted outcomes in deprived areas. General practice level data will be assessed to establish the profile of lipid management, compared with the regional and national levels. Health professionals involved in the organisation and delivery of routine lipid management to deprived populations will be interviewed to understand the implementation and delivery of current lipid management and associated challenges. The prototype intervention will be informed by the evidence generated from workpackages 1-3, which will be reviewed and assessed using the nominal group technique to reach consensus. Training and skills development materials will also be developed as needed.

Ethics and dissemination

Ethics approval has been obtained from the Faculty of Medical Sciences Research Ethics Committee at Newcastle University, UK. Findings will be disseminated to the participating sites, participants, commissioners, and in peer-reviewed journals and academic conferences.

For peer review only

For peer review only - http://bmjopen.bmj.com/site/about/guidelines.xhtml

Strengths and limitations of this study

- This study will develop a tailored lipid management intervention for deprived populations to help reduce health inequalities, using multiple methods.
- Multiple data sources will be used to assess and compare CVD risk management for deprived with non-deprived populations in Northern England to England overall.
- Primary care staff needs and challenges in delivering current lipid management and resources related to implementation will be identified.
- Some limitations to the study design include exclusion of non-English studies, publication bias, quality of data and selection bias in the rapid evidence review.

For peer review only

For peer review only - http://bmjopen.bmj.com/site/about/guidelines.xhtml

INTRODUCTION

Hyperlipidaemia contributes a significant proportion of modifiable cardiovascular disease (CVD) risk, which is the leading cause of mortality and morbidity in England and Europe [1 2], accounting for a third of deaths in the UK. Hyperlipidaemia, a high level of cholesterol or triglycerides in the blood, can be inherited, is often found in people who are overweight, have alcohol abuse or have an unhealthy diet [3]. Elevated levels of blood lipids represent a major risk factor for the development of coronary heart disease (CHD) and other cerebrovascular diseases including stroke, transient ischaemic attack (TIA), and peripheral arterial disease. People with a history of these events are also at increased risk of experiencing subsequent CVDs. Management of CVDs also places a significant economic burden on the National Health Service (NHS), with an estimated cost of £7.4 billion per annum [4].

Both national and international guidelines recommend the use of statins in people at risk of CVD [1 5]. Their use aims to reduce the synthesis of cholesterol, but evidence suggests that there is an underuse of lipid-lowering drugs among eligible patients [6 7]. CVD is also a condition that is strongly associated with health inequalities and disproportionally affects disadvantaged socioeconomic communities. People in disadvantaged socioeconomic groups experience a higher prevalence of CVD events but poorer outcomes and premature mortality, leading to the fact that people in the most deprived areas in England are four times more likely to die prematurely than those in the least deprived [8]. In addition, those living in socioeconomically disadvantaged neighbourhoods are found to have poor engagement with preventive health services, even though they are likely to benefit from screening and early treatment [9]. This may lead to an exacerbation of existing health inequalities. NICE guidance has recognised socioeconomic status as an additional factor that contributes to CVD risk. The NHS Long Term Plan [10] has also identified CVD as a clinical priority and stressed the wider impact on health inequalities, highlighting that heart disease-related mortality is the single largest contributor to the life expectancy gap between the most and least deprived. However, it failed to establish how health inequalities could be approached or addressed within local systems.

The North East of England is consistently ranked as having the highest poverty levels and the lowest health outcomes in England [11]. Scotland has established a programme to support general practices caring for the most deprived communities (the 'Deep End' project)[12] and, in early 2020, local GPs, Public Health leaders and academics collaborated to form a Deep End Steering Group for the North East and North Cumbria (NENC). Funding was then granted from the North East and North Cumbria Integrated Care System (NENC ICS) Prevention strand to establish and co-design a Deep End network for the region. The Deep End NENC network consists of 35 practices; practices identified as Deep End

For peer review only - http://bmjopen.bmj.com/site/about/guidelines.xhtml

are those that fall into the 10% most deprived practice populations in England. These practices have between 95.7% and 57.7% of registered patients living in the most deprived 15% of indices of multiple deprivation (IMD) data zones. Due to the high rates of long term conditions, unhealthy diets and physical inactivity, together with other competing priorities [13], people in areas of deprivation are likely to face greater challenges in managing CVDs. Ongoing effects from the pandemic are exacerbating these challenges and include difficulties attending review appointments in person, digital poverty impeding remote review, low levels of health literacy resulting in misunderstandings about the need to continue long term treatments, and closure of other support services [14], potentially widening health inequalities.

The NHS has set up the national CVD Prevention programme [15] which aims to develop targeted interventions to minimise risk factors by maximising diagnosis and treatment, accompanied by the GP contract to commission a new national CVD prevention audit for primary care [16]. However, no evidence is available on what and how the intervention has been implemented and for what health outcomes for deprived populations. There is therefore an urgent need to seek a theoretical underpinning to tailor the national programme in this context, which could support the CVD element of the NHS post-COVID-19 recovery plan with the region.

This study will examine the literature and practice-level data and undertake engagement with staff who provide primary care for deprived populations to define the components and mechanisms through which lipid management can be optimised to meet the identified needs. The study aims to 1) synthesise the evidence on interventions for deprived populations with CVDs or those with high risks and understand the outcomes associated with these interventions, 2) assess CVD risk management for deprived populations in the NENC in comparison with non-deprived populations in Northern England and with England overall in order to identify clinical gaps and needs, 3) investigate the implementation and delivery of current interventions for patients with CVDs and those with high risks, and 4) tailor and optimise the national prevention programme to suit the context and needs of deprived communities.

METHODS AND ANALYSIS

Study design

A mixed methods approach will be employed to inform the development of the intervention comprising a rapid review, a population based observational study and qualitative interviews. Four work packages (WPs) are proposed (Figure 1).

[insert Figure 1]

For peer review only - http://bmjopen.bmj.com/site/about/guidelines.xhtml

A project advisory group consisting of 6-8 members will be established to involve key opinion leaders across core fields, who will advise at each project stage, review intervention components for the consensus process and help disseminate the study outputs. Members will recruit from the Deep End network, ICS Prevention Board, Academic Health Science Network (AHSN) NENC lipid steering group, regional professional leads for lipid management, public members and academics with methodology expertise. This group will meet quarterly with the research team to oversee the execution of the study and provide advice and assistance.

Patient and public involvement

Patients' experiences are central to the research question and outcomes, although the focus of this project is on clinicians' experiences. The Deep End Steering group, consisting of local GPs, representatives from the North East Commissioning Support Unit (NECS), Newcastle University Medical School, Health Education England North East and the Postgraduate School of Primary Care, Directors of Public Health, NHS England, the Northern Cancer Alliance and local voluntary, community and social enterprise organisations, was consulted in order to shape the research focus and question, methods of data collection and dissemination. While the focus of the research is initially on clinicians' experience, the development of a patient and public involvement strategy was recognised in the consultation as an urgent requirement, and this is currently being developed. This results of this study will be widely shared via the Public Involvement and Community Engagement network for the National Institute for Health Research (NIHR) Applied Research Collaboration NENC.

WP1: Rapid review and logic model (months 1-3)

WP 1 will be a rapid review and synthesis of current evidence, aiming to identify evidence-based interventions for lipid management in deprived areas and targeted outcomes. The review will be conducted following Cochrane guidance on rapid reviews [17]. A logic model will be developed, informed by existing literature to describe how lipid management works in theory to benefit services and patients.

Type of studies

Empirical studies (i.e. original data collection) describing the setting, problem addressed, resource requirements, aim, intervention components, provider, method of delivery and objective and subjective outcomes will be included if conducted in an Organisation for Economic Co-operation and Development (OECD) country [18] (to ensure a degree of commonality in health system and socioeconomic and demographic context), published in peer-reviewed scientific journals, within the last 10 years (to mirror the NHS long term plan) and in the English language.

For peer review only - http://bmjopen.bmj.com/site/about/guidelines.xhtml

Type of participants

Studies that focus on people with disadvantaged socioeconomic status (education, income, occupation, social class, deprivation, poverty or an area-based proxy for deprivation derived from place of residence) will be included. Adults with CVD including angina, previous myocardial infarction (MI), revascularisation, stroke or TIA or symptomatic peripheral arterial disease, and those who do not have established CVD but are identified as having a high risk of developing CVDs [1] considering age, ethnicity, socioeconomic status, BMI, history of taking antihypertensive or lipid modification therapy, QRISK$\geq$10%, diabetes, nephropathy, familial hypercholesterolaemia or other inherited disorder of lipid metabolism, and other underlying medical conditions or treatment including people treated for HIV, with serious mental health problems, taking medicines that can cause dyslipidaemia or with autoimmune disorders.

Type of interventions

Multifaceted interventions delivered to deprived populations that aim to optimise care by maximising diagnosis and/or treatment to minimise individual risk factors will be considered.

Type of outcome measures

Studies with individual, area-based or both types of measures of socioeconomic deprivation will be included. This may be measured according to several characteristics including income, employment, education, disability, crime, housing and services and living environment deprivation. Because there is no universal recommendation for core outcome sets in studies on CVD prevention [19-21], studies will be eligible for inclusion regardless of outcomes measured or reported for health outcomes, this may include vascular-related outcomes, cognitive and functional outcomes, lifestyle, medical risk factors, cardioprotective medications and patient-reported outcome measures. Any measures of professionals', patients' and/or families' knowledge, attitudes or satisfaction will also be included.

Study identification

Cochrane CENTRAL, MEDLINE, PsycInfo, CINAHL will be searched for eligible studies. Detailed search strategies will be developed for each database. A preliminary search strategy developed for MEDLINE is designed by YF and validated by an information specialist (supplementary material 1). This search strategy was piloted in MEDLINE on 16th October 2021.

Study selection

Identified citations will be exported to Endnote X9 [22] for deduplication and screening. A random selection (10%) of study titles and abstracts will be screened independently by another researcher. Full text will be retrieved where citations appeared to meet the eligibility criteria or where a decision

For peer review only - http://bmjopen.bmj.com/site/about/guidelines.xhtml

to exclude will not be made on the information provided. Any discrepancies will be resolved by discussion with a third researcher.

Data extraction

Data will be extracted on author's first name, publication date, location (country in which the study was undertaken), study design, sample size, intervention details, control/comparison groups (if any), outcome measures and results, using a data extraction sheet that will be piloted on two retrieved study reports. Accuracy and consistency will be monitored through random double-extraction of 10% included studies by an independent researcher. Any discrepancies will be resolved by discussion with a third researcher. Where a study appears to have multiple citations, original authors will be contacted for clarification. All information from multiple citations will be used if no replies received.

Quality assessment

Quality appraisal of included studies will be performed using standardised tools adapted for purpose. Appropriate Critical Appraisal Skills Programme (CASP) tool will be used according to the study design, a random sample (10%) will be independently assessed by another researcher. Any discrepancies will be resolved by discussion with a third researcher.

Data synthesis

A narrative synthesis will be undertaken following Popay et al's [23] approach to conducting synthesis systematically and transparently. It will focus on the intervention components, effects of the interventions and mechanisms leading to the outcomes. Studies, interventions and outcomes will be examined and grouped according to the aim and components of the interventions. The variation on different characteristics of health systems will be taken into account when interpreting the intervention across OECD countries. A logic model will be produced to present context, intervention components and outcomes. Possible unintended adverse outcomes will also be provided.

WP2: Assessment and comparison of CVD risk management for deprived with non-deprived populations to England overall (months 2-4)

WP2 will be a population based observational study comparing retrospective data from practices in deprived communities in the NENC, practices in regional non-deprived communities and national practice-level data obtained from publicly accessible datasets and anonymised data requested from the NECS that securely house primary and secondary care datasets.

Data sources

The primary data source for this study will be the GP Practice Profiles [24] via Fingertips, a publicly accessible web tool containing national general practice profiles generated for all Quality and

For peer review only - http://bmjopen.bmj.com/site/about/guidelines.xhtml

Outcomes Framework (QOF)[25] 2019/20 with a list size of at least 750 patients. Available practice-level data include local demography, QOF domains and patient satisfaction. Other data sources used include the QOF, OpenPerscribing[26] and data requested from the NECS via Secondary Uses Services (SUS)[27] data (Table 1).

Table 1 Data sources and variables

Data source	Description	Level of data available	Variables and variable description
GP Practice Profiles	Date reported by GPs to the NHS that refers to all patients in a practice	• Individual practice	• Practice size<br>• Mean practice age<br>• Deprivation score<br>• Age groups<br>• Percentage of patients positive experiences as "good"<br>• Percentage of practice access rated by patients as "good"<br>• Percentage with a long term condition<br>• Education status<br>• Working status<br>• Life expectancy by sex
QOF	An indication of the overall achievement of a practice through a points system, concerning clinical, public health, public health – additional services, and quality improvement.<br>It also has cardiovascular group data.	• Individual practice	• QOF score<br>• Total on the atrial fibrillation (AF) register, prevalence<br>• Total on the CVD-primary prevention (CVD-PP) register, prevalence<br>• Total on the CHD register, prevalence<br>• Total on the heart failure (HF) register, prevalence<br>• Total on the left ventricular systolic dysfunction (LVSD) register, prevalence<br>• Total on the hypertension (HYP) register, prevalence<br>• Total on the peripheral arterial disease (PAD) register, prevalence<br>• Total on the stroke and transient ischaemic attack (STIA) register, prevalence<br>•
OpenPrescribing	Imports national prescribing data published by NHS Business Services Authority	• Individual practice<br>• CCG level	• Total statin<br>• Total low and medium intensity statin

For peer review only - http://bmjopen.bmj.com/site/about/guidelines.xhtml

Study population

The study population are patients aged 16 and above who have registered with the 34 Deep End practices in the NENC and have been diagnosed with any form of CVDs recorded on the QOF from 2019 to 2020. The study comparators are the patients registered in non-Deep End practices in the region and all registered patients in England where data is available. Data is aggregated to the GP practice level in which variables will be summarised if they are at the patient level.

Data analysis

Descriptive analysis will be used to provide a quick and low cost approach to assess CVD risk management and give descriptive statistics or investigate relationships between factors. GP practice code will be used to link data across all datasets. Due to the nature of the aggregated data available from the public sources used (Fingertips[24] and QOF), it will be not possible to control any of the comparisons for age, gender, deprivation or ethnicity. Descriptive statistics, using means, standard deviations, and range, will be used to compare the practice profile of the 34 Deep End practices with non-Deep End in the region and England average level. The prevalence of risk factors and statin prescribing will be analysed with an appropriate statistical test (i.e., two-sample t-test, single sample t-test, and paired t-test), which will yield p values that indicate the statistical significance of any differences between Deep End, non-Deep End and England level. A paired t-test will be used to understand whether there was a difference in outcomes before and at the time of the COVID-19 pandemic. Confidence intervals for differences in means, medians or percentages will be calculated. All significance tests will be performed at the 5% level. Stata version 16 will be used to facilitate data analysis.

WP3: Interviews with health professionals (months 3-8)

WP3 will be qualitative interviews with staff involved in the organisation and delivery of routine lipid management in practices that are part of the Deep End Network. The aim of the interviews will be to understand the implementation and delivery of current lipid management and identify their needs and challenges.

Participants and recruitment

All health professionals involved in the management of CVDs in the practice are eligible to take part including GPs, pharmacists, assistant practitioners, practice nurses and social prescribers.

Study recruitment will be supported by the Deep End practice Network, who will send an email containing brief study information to healthcare professionals working in participating practices. Health professionals can express their interests by responding to the email straight to the research

For peer review only - http://bmjopen.bmj.com/site/about/guidelines.xhtml

team. A reminder will be sent to those who have not responded in two weeks. Maximum variation sampling will be used to ensure a broad representation of health professionals on dimensions including job titles/roles, grade, speciality, length of working, and demographics. Reasons given by practices for declining to participate will be recorded to inform feasibility assessment to further studies.

Data collection

With participants' informed written consent, semi-structured interviews will be conducted via telephone or online (i.e. Zoom or MS Teams) for up to 60 mins. A topic guide was drafted to address the research questions and piloted with two primary care health professionals to ensure the questions prepared are relevant for the context and acceptable. Questions considered important but not originally included were also sought from the pilot interviews, and the topic guide was amended accordingly (supplementary material 2). As interviews continue, the topic guide will also allow a deeper exploration of emerging themes and participants' feedback, while maintaining a consistent core of questions. Data collection will end when data saturation is reached indicating no new information is discovered.

Data analysis

Interviews will be digitally recorded (with consent), transcribed and data managed using NVivo 12, a qualitative software programme to assist with the organisation and coding of data. Data will be analysed using Framework Analysis, which provides a systematic approach to sifting, charting, and sorting material using the key themes and issues. Initial line-by-line coding will be undertaken. The connections and relationships of these codes will be explored, contributing to the development of themes. An analytical framework will have been developed as the coding process progresses and themes emerges. Codes and themes from each transcript will be compared and integrated using the constant comparison process, enabling continuous updates on the interview topic guide and the thorough interpretation of the study data. To ensure trustworthiness and rigour of the analysis, the coding framework will be developed and assured by double coding of a random sample of transcripts (10%) as a validity check and exploring alternative interpretations of the data.

WP4: Intervention development (months 7-9)

WP 4 will develop the prototype of the intervention in collaboration with the Academic Health Science Network NENC which delivers the CVD Prevention programme, part of which includes a national programme mandated by NHS England and NHS Improvement.

For peer review only - http://bmjopen.bmj.com/site/about/guidelines.xhtml

Guided by the Medical Research Council (MRC) Framework for developing and evaluating complex interventions [28], the development of intervention will be informed by integrating the outcomes of the literature evidence, current CVD management profile, and stakeholder engagement undertaken in WPs1-3, in an iterative and progressive approach. The national programme and its key components will be examined against the gaps, needs, and challenges identified to consider the wider context. The prototype intervention will be designed taking account of health professionals' existing commitments in these practices and challenging working environments. Training and skills development materials for health professionals will also be developed to facilitate them in delivering the tailored intervention. The logic model produced in WP1 will be refined to map key intervention processes and outcomes.

Design

The prototype intervention will be reviewed and assessed by the project advisory group, guided by a nominal group technique, a consensus method that allows for the generation of views and thoughts from group participants whilst maintaining anonymity throughout [29].

Data collection

The group will be provided with details of the interventions and refined logic model, to seek further comments and explore if the intervention is feasible, acceptable, and implementable in the context. The APEASE criteria [30] will be used to determine the acceptability, practicability, effectiveness, affordability, side-effects, and equity aspects of the intervention. The nominal group technique will involve two main sections:

1. The group will be asked to provide their comments on the intervention, training materials, and logic model. All comments will be collated and grouped into main themes for each member to rate their top 10 priorities of the comments. Group ratings will be summated, and the group's collective top 10 priorities will be presented to the group and discussed.
2. Each will re-rate the group's top 10 priorities and provide a weighting for their top 10 comments in the scale ranging from 1=least important to 100=most important. These weightings will be summated after the meeting, which will be used to refine the intervention and the logic model. The refined version will be sent out to each member for further comments. It is expected that this process will be repeated twice until a census is reached.

Data analysis

The initial listing of comments, clarification, and discussion of comments in section 1 will be analysed thematically, with further discussion with the research team. The scale data generated in section 2 will be averaged so that the comments can be re-ordered according to weighted ranked priority. The

For peer review only - http://bmjopen.bmj.com/site/about/guidelines.xhtml

individual/group rankings produced in sections 1 and 2 will be compared to estimate the level of agreement between the sections and to observe the process of reaching consensus. Firstly, these comparisons will be made by calculating the percentage agreement between the sections, in terms of the comments that appear in the top ten priorities each time. Secondly, the movement in ranking between sections 1 and 2 will be estimated using Cohen's kappa statistic of chance-corrected agreement [31]. A kappa value of >0.40 is considered to represent a moderate level of agreement [32].

ETHICS AND DISSEMINATION

Ethics approval has been sought from the Faculty of Medical Sciences Research Ethics Committee at Newcastle University (reference no.: 2209/14251) UK, a letter of support has also been issued by the NENC Deep End Network.

Dissemination will be led by the research team and supported by the project advisory group. Reports will be produced and shared with the NENC ICS Prevention Board, Inequalities Board, Deep End network, National Institute for Health Research (NIHR) Applied Research Collaboration (ARC) NENC, and AHSN NENC. The findings will be disseminated to the participating sites, participants, commissioners, and in peer-reviewed journals and academic conferences.

For peer review only - http://bmjopen.bmj.com/site/about/guidelines.xhtml

FIGURES

Figure 1 Study design and related work packages

ACKNOWLEDGEMENT

We thank NENC Deep End Steering Group and NECSU for their support in this study.

AUTHORS' CONTRIBUTIONS

YF, JN and PW conceived the study design. YF drafted and revised the manuscript and obtained the ethics approval. EYHT, SS, JN and PW independently reviewed and contributed to revising and approving the final version.

FUNDING

This project is supported by the National Institute of Health Research (NIHR) [Applied Research Collaboration North East and North Cumbria (NIHR200173)]. The views expressed are those of the author(s) and not necessarily those of the NIHR or the Department of Health and Social Care.

EYHT (National Institute for Health Research (NIHR) Clinical Lecturer) is funded by the NIHR. The views expressed in this publication are those of the author(s) and not necessarily those of the NIHR, NHS or the UK Department of Health and Social Care.

COMPETING INTERESTS

None declared.

For peer review only

REFERENCES

1. NICE. Cardiovascular disease: risk assessment and reduction, including lipid modification National Institute for Health and Care Excellence; 2014 [accessed 19th July 2021.
2. Eurostat. Cardiovascular diseases statistics: Eurostat; 2020 [Available from: https://ec.europa.eu/eurostat/statistics-explained/index.php?title=Cardiovascular_diseases_statistics#Deaths_from_cardiovascular_diseases accessed 20th July 2021.
3. Nelson RH. Hyperlipidemia as a risk factor for cardiovascular disease. *Prim Care* 2013;40(1):195-211. doi: 10.1016/j.pop.2012.11.003 [published Online First: 2012/12/04]
4. Public Health England. Health matters: preventing cardiovascular disease: Public Health England; 2019 [Available from: https://www.gov.uk/government/publications/health-matters-preventing-cardiovascular-disease/health-matters-preventing-cardiovascular-disease accessed 20th July 2021.
5. Mach F, Baigent C, Catapano AL, et al. 2019 ESC/EAS Guidelines for the management of dyslipidaemias: lipid modification to reduce cardiovascular risk: The Task Force for the management of dyslipidaemias of the European Society of Cardiology (ESC) and European Atherosclerosis Society (EAS). *European Heart Journal* 2019;41(1):111-88. doi: 10.1093/eurheartj/ehz455
6. Wu J, Zhu S, Yao GL, et al. Patient Factors Influencing the Prescribing of Lipid Lowering Drugs for Primary Prevention of Cardiovascular Disease in UK General Practice: A National Retrospective Cohort Study. *PLOS ONE* 2013;8(7):e67611. doi: 10.1371/journal.pone.0067611
7. Ray KK, Molemans B, Schoonen WM, et al. EU-Wide Cross-Sectional Observational Study of Lipid-Modifying Therapy Use in Secondary and Primary Care: the DA VINCI study. *European Journal of Preventive Cardiology* 2020 doi: 10.1093/eurjpc/zwaa047
8. NHS. Optimising treatment of high-risk conditions: NHS; 2021 [Available from: https://www.england.nhs.uk/ltphimenu/cvd/optimising-treatment-of-high-risk-conditions/ accessed 28th September 2021.
9. Lang S-J, Abel GA, Mant J, et al. Impact of socioeconomic deprivation on screening for cardiovascular disease risk in a primary prevention population: a cross-sectional study. *BMJ Open* 2016;6(3):e009984. doi: 10.1136/bmjopen-2015-009984
10. NHS. The NHS Long Term Plan London: National Health Service; 2019 [Available from: https://www.longtermplan.nhs.uk/wp-content/uploads/2019/08/nhs-long-term-plan-version-1.2.pdf accessed 20th July 2021.
11. Corris V, Dormer E, Brown A, et al. Health inequalities are worsening in the North East of England. *British Medical Bulletin* 2020;134(1):63-72.
12. Watt G, Brown G, Budd J, et al. General Practitioners at the Deep End: The experience and views of general practitioners working in the most severely deprived areas of Scotland. *Occasional paper (Royal College of General Practitioners)* 2012(89):i.
13. Holmes J. Tackling obesity: the role of the NHS in a whole-system approach: The King's Fund; 2021 [Available from: https://www.kingsfund.org.uk/sites/default/files/2021-07/Tackling%20obesity.pdf accessed 20th July 2021.
14. Norman C, Wildman JM, Sowden S. COVID-19 at the Deep End: A Qualitative Interview Study of Primary Care Staff Working in the Most Deprived Areas of England during the COVID-19 Pandemic. *International Journal of Environmental Research and Public Health* 2021;18(16):8689.
15. NHS England. Cardiovascular disease London: National Health Service,; 2019 [Available from: https://www.england.nhs.uk/ourwork/clinical-policy/cvd/ accessed 20th July 2021.
16. NHS England. CVDPREVENT London: National Health Service; 2019 [Available from: https://www.england.nhs.uk/ourwork/clinical-policy/cvd/cvdprevent/ accessed 20th July 2021.

For peer review only - http://bmjopen.bmj.com/site/about/guidelines.xhtml

17. Garritty C, Gartlehner G, Kamel C, et al. Cochrane Rapid Reviews Interim Guidance from the Cochrane Rapid Reviews Methods Group. March 2020, 2020.
18. Reibling N, Ariaans M, Wendt C. Worlds of Healthcare: A Healthcare System Typology of OECD Countries. *Health Policy* 2019;123(7):611-20. doi: https://doi.org/10.1016/j.healthpol.2019.05.001
19. McNamara RL, Spatz ES, Kelley TA, et al. Standardized Outcome Measurement for Patients With Coronary Artery Disease: Consensus From the International Consortium for Health Outcomes Measurement (ICHOM). *Journal of the American Heart Association* 2015;4(5):e001767. doi: doi:10.1161/JAHA.115.001767
20. Neilson LM, Swart ECS, Good CB, et al. Identifying Outcome Measures for Coronary Artery Disease Value-Based Contracting Using the Delphi Method. *Cardiology and Therapy* 2019;8(1):135-43. doi: 10.1007/s40119-019-0132-7
21. Canavan M, Smyth A, Robinson SM, et al. Attitudes to outcomes measured in clinical trials of cardiovascular prevention. *QJM: An International Journal of Medicine* 2015;109(6):391-97. doi: 10.1093/qjmed/hcv132
22. Hupe M. EndNote X9. *Journal of Electronic Resources in Medical Libraries* 2019;16(3-4):117-19. doi: 10.1080/15424065.2019.1691963
23. Popay J, Roberts H, Sowden A, et al. Guidance on the conduct of narrative synthesis in systematic reviews. *A product from the ESRC methods programme Version* 2006;1:b92.
24. Public Health England. Public Health Profiles.: Public Health England; 2021 [Available from: https://fingertips.phe.org.uk accessed 29th Oct 2021.
25. NHS Digital. Quality Outcomes Framework (QOF): NHS Digital; 2021 [accessed 29th October 2021.
26. OpenPrescribing.net. EBM DataLab, University of Oxford, 2017.
27. NHS Digital. Secondary Uses Service (SUS): NHS Digital; 2021 [Available from: https://digital.nhs.uk/services/secondary-uses-service-sus accessed 29th Oct 2021.
28. Craig P, Dieppe P, Macintyre S, et al. Developing and evaluating complex interventions: the new Medical Research Council guidance. *Bmj* 2008;337
29. Harvey N, Holmes CA. Nominal group technique: an effective method for obtaining group consensus. *International journal of nursing practice* 2012;18(2):188-94.
30. West R, Michie S, Atkins L, et al. Achieving behaviour change: A guide for local government and partners London: Public Health England; 2019 [Available from: https://assets.publishing.service.gov.uk/government/uploads/system/uploads/attachment_data/file/875385/PHEBI_Achieving_Behaviour_Change_Local_Government.pdf accessed 14th September 2021.
31. Cohen J. A coefficient of agreement for nominal scales. *Educational and Psychological Measurement* 1960
32. Landis JR, Koch GG. The measurement of observer agreement for categorical data. *biometrics* 1977:159-74.

For peer review only

For peer review only - http://bmjopen.bmj.com/site/about/guidelines.xhtml

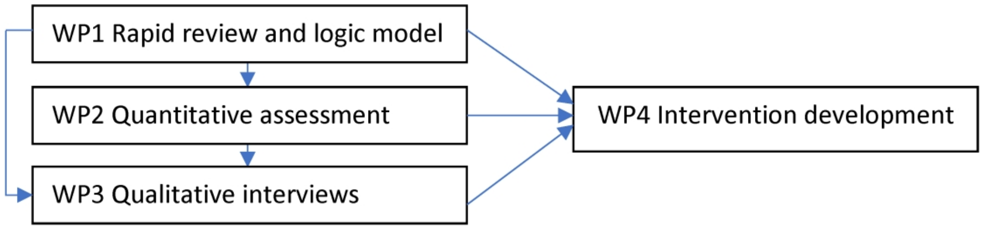

Figure 1 Study design and related work packages

139x34mm (600 x 600 DPI)

Supplementary Material 1 Search strategy for MEDLINE (Ovid) (1996 to October Week 4 2021)

1. exp Cardiovascular Diseases/
2. cardio*.tw.
3. cardia*.tw.
4. heart*.tw.
5. coronary*.tw.
6. angina*.tw.
7. ventric*.tw.
8. myocard*.tw.
9. pericard*.tw.
10. isch?em*.tw.
11. emboli*.tw.
12. arrhythmi*.tw.
13. thrombo*.tw.
14. atrial fibrillat*.tw.
15. tachycardi*.tw.
16. endocardi*.tw.
17. (sick adj sinus).tw.
18. exp Stroke/

For peer review only

For peer review only - http://bmjopen.bmj.com/site/about/guidelines.xhtml

19. (stroke or strokes).tw.
20. cerebrovasc*.tw.
21. cerebral vascular.tw.
22. apoplexy.tw.
23. ((brain* or cerebral or lacunar) adj2 infarct*).tw.
24. exp Hypertension/
25. hypertensi*.tw.
26. peripheral arter* disease*.tw.
27. ((high or increased or elevated) adj2 blood pressure).tw.
28. exp Hyperlipidemias/
29. hyperlipid*.tw.
30. hyperlip?emia*.tw.
31. hypercholesterol*.tw.
32. hypercholester?emia*.tw.
33. hyperlipoprotein?emia*.tw.
34. hypertriglycerid?emia*.tw.
35. exp Arteriosclerosis/
36. exp Cholesterol/
37. cholesterol.tw.
38. Blood Pressure/

For peer review only

39. 1 or 2 or 3 or 4 or 5 or 6 or 7 or 8 or 9 or 10 or 11 or 12 or 13 or 14 or 15 or 16 or 17 or 18 or 19 or 20 or 21 or 22 or 23 or 24 or 25 or 26 or 27 or 28 or 29 or 30 or 31 or 32 or 33 or 34 or 35 or 36 or 37 or 38
40. exp Socioeconomic Factors/
41. exp social class/
42. socioecon*.tw.
43. demographic*.tw.
44. disadvantage*.tw.
45. disparit*.tw.
46. deprivation.tw.
47. exp Health Services Accessibility/
48. deprive*.tw.
49. pover*.tw.
50. inequalit*.tw.
51. education*.tw.
52. unemploy*.tw.
53. employed.tw.
54. employment.tw.
55. income.tw.
56. occupation*.tw.
57. SES.tw.

For peer review only

58. class.tw.

59. economic.tw.

60. (social adj1 (class or factor or factors)).tw.

61. 40 or 41 or 42 or 43 or 44 or 45 or 46 or 47 or 48 or 49 or 50 or 51 or 52 or 53 or 54 or 55 or 56 or 57 or 58 or 59 or 60

62. exp General Practitioners/

63. exp General Practice/

64. general pract*.tw.

65. exp Family Practice/

66. family pract*.tw.

67. family medicine/

68. exp Family Practice/

69. exp Physicians, Family/

70. family phys*.tw.

71. exp Nurse Practitioners/

72. exp Nurse Clinicians/

73. nurse pract*.tw.

74. nurse clinic*.tw.

75. exp Primary Health Care/

76. primary care.tw.

77. exp Community Health Services/

For peer review only - http://bmjopen.bmj.com/site/about/guidelines.xhtml

78. community care.tw.

79. 62 or 63 or 64 or 65 or 66 or 67 or 68 or 69 or 70 or 71 or 72 or 73 or 74 or 75 or 76 or 77 or 78

80. 39 and 61 and 79

81. (Algeria$ or Egypt$ or Liby$ or Morocc$ or Tunisia$ or Western Sahara$ or Angola$ or Benin or Botswana$ or Burkina Faso or Burundi or Cameroon or Cape Verde or Central African Republic or Chad or Comoros or Congo or Djibouti or Eritrea or Ethiopia$ or Gabon or Gambia$ or Ghana or Guinea or Keny$ or Lesotho or Liberia or Madagasca$ or Malawi or Mali or Mauritania or Mauritius or Mayotte or Mozambiq$ or Namibia$ or Niger or Nigeria$ or Reunion or Rwand$ or Saint Helena or Senegal or Seychelles or Sierra Leone or Somalia or South Africa$ or Sudan or Swaziland or Tanzania or Togo or Ugand$ or Zambia$ or Zimbabw$ or China or Chinese or Hong Kong or Macao or Mongolia$ or Taiwan$ or Belarus or Moldov$ or Russia$ or Ukraine or Afghanistan or Armenia$ or Azerbaijan or Bahrain or Cyprus or Cypriot or Georgia$ or Iran$ or Iraq$ or Israel$ or Jordan$ or Kazakhstan or Kuwait or Kyrgyzstan or Leban$ or Oman or Pakistan$ or Palestin$ or Qatar or Saudi Arabia or Syria$ or Tajikistan or Turkmenistan or United Arab Emirates or Uzbekistan or Yemen or Bangladesh$ or Bhutan or British Indian Ocean Territory or Brunei Darussalam or Cambodia$ or India$ or Indonesia$ or Lao or People's Democratic Republic or Malaysia$ or Maldives or Myanmar or Nepal or Philippin$ or Singapore or Sri Lanka or Thai$ or Timor Leste or Vietnam or Albania$ or Andorra or Bosnia$ or Herzegovina$ or Bulgaria$ or Croatia$ or Estonia or Faroe Islands or Greenland or Liechtenstein or Lithuani$ or Macedonia or Malta or maltese or Romania or Serbia$ or Montenegro or Slovenia or Svalbard or Argentina$ or Belize or Bolivia$ or Brazil$ or chile or Chilean or Colombia$ or Costa Rica$ or Cuba or Ecuador or El Salvador or French Guiana or Guatemala$ or Guyana or Haiti or Honduras or Jamaica$ or Nicaragua$ or Panama or Paraguay or Peru or Puerto Rico or Suriname or Uruguay or Venezuela or developing countr$ or south America$).ti,sh. [mp=title, abstract, original title, name of substance word, subject heading word, floating sub-heading word, keyword heading word, organism supplementary concept word, protocol supplementary concept word, rare disease supplementary concept word, unique identifier, synonyms]

82. 80 not 81

83. limit 82 to (abstracts and english language and humans and yr="2011 -Current")

For peer review only

Interview topic guide

This interview is to learn from your experience on what and how lipid management is currently delivered to patients in your practice. The aim is to understand the implementation process and resources available for the existing interventions targeting patients with cardiovascular disease (CVD) and/or risks, as well as your needs and any challenges to providing lipid management optimisation in deprived communities.

The interview will be recorded with your permission. It will last for about 45-60 minutes and any information you give will be kept confidential and anonymous.

Our conversation will cover:

- Your job title, an overview of patients in your practice, what your role involves for patients with CVD risks;
- Your understanding of lipid management and what it involves;
- How patients with CVD (or CVD risks) are managed and their clinical pathways in your practice (screening/assessment/monitoring/treatment/outcomes/referrals):
    - How you identify patients with CVD risks (e.g. NHS Health Check; frequency), and is there anything else that would indicate the need for care for you;
    - What assessment tool(s) you use to understand their needs;
    - How you monitor their disease progress;
    - Existing treatment and care provided for patients and rationale for that;
    - Clinically meaningful outcomes;
    - How and when you decide on referrals and for what services.
- Your views on the implementation process of current interventions targeting patients with CVD in your practice, and what resource is needed for their delivery;
- Your experience/challenges of managing patients' needs, and factors that influence the delivery and quality of lipid management to patients;
- The impact of COVID on lipid management in your practice and solutions implemented;
- To what extent you have accessed training (general/lipid) to support your clinical role; other information, skills and training that you may need to help you optimise lipid management;
- Any other questions or comments and suggestions that you want to mention on how you provide services for patients with CVD and what additional support you might need;
- Any other questions you have about this study.

For peer review only

For peer review only - http://bmjopen.bmj.com/site/about/guidelines.xhtml

PRISMA-P (Preferred Reporting Items for Systematic review and Meta-Analysis Protocols) 2015 checklist: recommended items to address in a systematic review protocol*

Section and topic	Item No	Checklist item	Page no.
**ADMINISTRATIVE INFORMATION**			
Title:			
Identification	1a	Identify the report as a protocol of a systematic review	
Update	1b	If the protocol is for an update of a previous systematic review, identify as such	
Registration	2	If registered, provide the name of the registry (such as PROSPERO) and registration number	
Authors:			
Contact	3a	Provide name, institutional affiliation, e-mail address of all protocol authors; provide physical mailing address of corresponding author	
Contributions	3b	Describe contributions of protocol authors and identify the guarantor of the review	
Amendments	4	If the protocol represents an amendment of a previously completed or published protocol, identify as such and list changes; otherwise, state plan for documenting important protocol amendments	
Support:			
Sources	5a	Indicate sources of financial or other support for the review	
Sponsor	5b	Provide name for the review funder and/or sponsor	
Role of sponsor or funder	5c	Describe roles of funder(s), sponsor(s), and/or institution(s), if any, in developing the protocol	
**INTRODUCTION**			
Rationale	6	Describe the rationale for the review in the context of what is already known	5-6
Objectives	7	Provide an explicit statement of the question(s) the review will address with reference to participants, interventions, comparators, and outcomes (PICO)	6
**METHODS**			
Eligibility criteria	8	Specify the study characteristics (such as PICO, study design, setting, time frame) and report characteristics (such as years considered, language, publication status) to be used as criteria for eligibility for the review	7-8
Information sources	9	Describe all intended information sources (such as electronic databases, contact with study authors, trial registers or other grey literature sources) with planned dates of coverage	8
Search strategy	10	Present draft of search strategy to be used for at least one electronic database, including planned limits, such that it could be	Supplementary

For peer review only

		repeated	
Study records:			
Data management	11a	Describe the mechanism(s) that will be used to manage records and data throughout the review	8
Selection process	11b	State the process that will be used for selecting studies (such as two independent reviewers) through each phase of the review (that is, screening, eligibility and inclusion in meta-analysis)	8
Data collection process	11c	Describe planned method of extracting data from reports (such as piloting forms, done independently, in duplicate), any processes for obtaining and confirming data from investigators	8
Data items	12	List and define all variables for which data will be sought (such as PICO items, funding sources), any pre-planned data assumptions and simplifications	9
Outcomes and prioritization	13	List and define all outcomes for which data will be sought, including prioritization of main and additional outcomes, with rationale	9
Risk of bias in individual studies	14	Describe anticipated methods for assessing risk of bias of individual studies, including whether this will be done at the outcome or study level, or both; state how this information will be used in data synthesis	8-9
Data synthesis	15a	Describe criteria under which study data will be quantitatively synthesised	9
	15b	If data are appropriate for quantitative synthesis, describe planned summary measures, methods of handling data and methods of combining data from studies, including any planned exploration of consistency (such as I^2, Kendall's τ)	
	15c	Describe any proposed additional analyses (such as sensitivity or subgroup analyses, meta-regression)	
	15d	If quantitative synthesis is not appropriate, describe the type of summary planned	
Meta-bias(es)	16	Specify any planned assessment of meta-bias(es) (such as publication bias across studies, selective reporting within studies)	n/a
Confidence in cumulative evidence	17	Describe how the strength of the body of evidence will be assessed (such as GRADE)	n/a

*** It is strongly recommended that this checklist be read in conjunction with the PRISMA-P Explanation and Elaboration (cite when available) for important clarification on the items. Amendments to a review protocol should be tracked and dated. The copyright for PRISMA-P (including checklist) is held by the PRISMA-P Group and is distributed under a Creative Commons Attribution Licence 4.0.**

From: Shamseer L, Moher D, Clarke M, Ghersi D, Liberati A, Petticrew M, Shekelle P, Stewart L, PRISMA-P Group. Preferred reporting items for systematic review and meta-analysis protocols (PRISMA-P) 2015: elaboration and explanation. BMJ. 2015 Jan 2;349(jan02 1):g7647.

For peer review only
